# Short-read whole genome sequencing identifies causative variants in most individuals with previously unexplained aniridia

**DOI:** 10.1136/jmg-2023-109181

**Published:** 2023-11-30

**Authors:** Hildegard Nikki Hall, David Parry, Mihail Halachev, Kathleen A Williamson, Kevin Donnelly, Jose Campos Parada, Shipra Bhatia, Jeffrey Joseph, Simon Holden, Trine E Prescott, Pierre Bitoun, Edwin P Kirk, Ruth Newbury-Ecob, Katherine Lachlan, Juan Bernar, Veronica van Heyningen, David R FitzPatrick, Alison Meynert

**Affiliations:** 1 Institute of Genetics and Cancer, The University of Edinburgh MRC Human Genetics Unit, Edinburgh, UK; 2 Illumina United Kingdom, Edinburgh, UK; 3 MRC Human Genetics Unit, The University of Edinburgh, Edinburgh, UK; 4 East Anglia Regional Genetics Service, Addenbrooke’s Hospital, Cambridge, UK; 5 Department of Medical Genetics, Telemark Hospital, Skien, Norway; 6 Consultations de Génétique médicale, Service de Pédiatrie, CHU Paris-Nord, Hôpital Jean Verdier, Bondy, France; 7 Centre for Clinical Genetics, Sydney Children’s Hospital Randwick, Randwick, New South Wales, Australia; 8 Department of Clinical Genetics, University Hospitals Bristol NHS Foundation Trust, Bristol, UK; 9 University Hospital Southampton, NHS Foundation Trust Wessex Clinical Genetics Service, Southampton, UK; 10 Department of Genetics, Hospital Ruber Internacional, Madrid, Spain; 11 Institute of Ophthalmology, University College London, London, UK

## Abstract

**Background:**

Classic aniridia is a highly penetrant autosomal dominant disorder characterised by congenital absence of the iris, foveal hypoplasia, optic disc anomalies and progressive opacification of the cornea. >90% of cases of classic aniridia are caused by heterozygous, loss-of-function variants affecting the *PAX6* locus.

**Methods:**

Short-read whole genome sequencing was performed on 51 (39 affected) individuals from 37 different families who had screened negative for mutations in the *PAX6* coding region.

**Results:**

Likely causative mutations were identified in 22 out of 37 (59%) families. In 19 out of 22 families, the causative genomic changes have an interpretable deleterious impact on the *PAX6* locus. Of these 19 families, 1 has a novel heterozygous *PAX6* frameshift variant missed on previous screens, 4 have single nucleotide variants (SNVs) (one novel) affecting essential splice sites of *PAX6* 5′ non-coding exons and 2 have deep intronic SNV (one novel) resulting in gain of a donor splice site. In 12 out of 19, the causative variants are large-scale structural variants; 5 have partial or whole gene deletions of *PAX6*, 3 have deletions encompassing critical *PAX6 cis*-regulatory elements, 2 have balanced inversions with disruptive breakpoints within the *PAX6* locus and 2 have complex rearrangements disrupting *PAX6*. The remaining 3 of 22 families have deletions encompassing *FOXC1* (a known cause of atypical aniridia). Seven of the causative variants occurred *de novo* and one cosegregated with familial aniridia. We were unable to establish inheritance status in the remaining probands. No plausibly causative SNVs were identified in *PAX6 cis*-regulatory elements.

**Conclusion:**

Whole genome sequencing proves to be an effective diagnostic test in most individuals with previously unexplained aniridia.

## Introduction

Historically the molecular genetic investigation of Mendelian disorders has focused on sequencing of the coding regions of causative genes often in combination with genomic copy number analysis. Depending on the phenotypic specificity of the disease under investigation, these tests could involve sequencing of a single gene, a panel of genes or whole exome analysis. One of the motivations for restricting diagnostic analysis to the coding regions of genes has been the availability of well-characterised and validated approaches to predict the consequence of each variant and to assign a confidence to its pathogenicity.^1^ ^2^

The wider adoption of whole genome sequencing (WGS) as a diagnostic tool,^3^ together with the guidelines that aim to standardise the interpretation of variants outside the coding regions of genes, provides an opportunity to increase the utility of diagnostic genetic analyses.^4^ Here we have used short-read WGS to try to identify causative variants in individuals with aniridia in whom no diagnosis was found by prior molecular genetic testing approaches. Classic aniridia has major advantages in such a study as a Mendelian disease in which the phenotype in early childhood (congenital absence of the iris with foveal hypoplasia) has a high (~0.9) positive predictive value for detecting a heterozygous loss-of-function mutation at a single locus (*PAX6*).^5^

*PAX6* encodes a dosage critical transcription factor that is essential for vertebrate eye and brain development,^6^ and many *cis*-regulatory elements (CRE) controlling its expression during development have been functionally characterised.^7^ ^8^ Diagnostic analysis of this locus has been in routine clinical use now for 30 years, providing a very large data set of causative variants and established disease mechanisms.^5^

As this study will show, WGS is able, with reasonable sensitivity, to identify causative variants in individuals with a clinical diagnosis of aniridia who have previously, often repeatedly, tested negative for mutations in the coding region of *PAX6*.

## Methods

### Clinical research participants

This project used clinical information and biological samples from individuals referred to the Medical Research Council (MRC) Human Genetics Unit Eye Malformations Study. All affected individuals had classical aniridia; those with significant additional ocular phenotypes were excluded, such as severe microphthalmia or severe congenital corneal opacification. Pseudonymised research participant identifiers (RPIDs), relevant clinical features and molecular analyses performed prior to this study are provided in table 1.

### Preparation of genomic DNA and quality control

The quality and concentration of patient genomic DNA (gDNA) samples were assessed by agarose gel electrophoresis, NanoDrop 1000 spectrophotometry (Thermo Fisher Scientific, Inchinnan, UK), and/orQubit 3 fluorometer high sensitivity (HS) assay (Invitrogen, Thermo Fisher Scientific). In the case of one family trio, the DNA for the parents was extracted from patient-derived lymphoblastoid cell lines (LCLs). All probands had had prior Sanger sequencing of the coding regions of *PAX6* and *MAB21L1*.

### Whole genome sequencing

WGS was performed by BGI (New Territories, Hong Kong) for 9 samples and Edinburgh Genomics (Edinburgh, UK) for the remaining 42 samples.

Detailed methods for massively parallel sequencing library preparation are found in online supplemental information. In brief, gDNA was sheared using a Covaris ultrasonicator, and the fragments were A-tailed, size selected and adaptor ligated prior to PCR amplification. Libraries were clustered onto a flow cell for sequencing using HiSeqX (Illumina).

### WGS mapping, alignment, quality control and single nucleotide variant/INDEL variant calling

WGS samples were processed with Bcbio V.0.9.7, which uses BWA V.0.7.13^9^ to align reads to the human reference genome assembly hg38, samblaster V.0.1.22^10^ to mark duplicates, and GATK V.3.4.0^11^ to realign small insertions and deletions (INDELs) and recalibrate base quality scores. Families and singletons were genotyped following GATK best practices^12^ using V.4.0.2.1 of the toolkit. HaplotypeCaller was used to generate GVCFs, which were imported into a database via GenomicsD-BImport and genotyped with GenotypeGVCFs. Variant quality score recalibration was carried out with GATK VariantRecalibrator and ApplyVQSR separately for single nucleotide variants (SNVs) and INDELs. Low-quality (GQ <20) genotypes were filtered.

### Structural variant calling

#### IGV visualisation

Direct inspection of the aligned WGS data was performed to detect structural variant (SV) at known aniridia loci, using IGV (Integrative Genomics Viewer, Broad Institute, Massachusetts, USA),^13^ via visualisation of breakpoints and coverage. The gene and regulatory regions of *PAX6* were examined in all cases. Where *PAX6* was negative, *FOXC1* and *PITX2* were included with their regulatory regions, and *MAB21L1*; in some cases, this was expanded to additional loci (*FOXE3, RARB, ADAMTSL1, CYP1B1*); for a list of coordinates, see online supplemental table S1. In IGV, reads were coloured both by insert size (to detect breakpoints of deletions/insertions) and by pair orientation (to detect breakpoints of chromosomal rearrangements such as inversions).

#### Bioinformatic SV calling

CNVs were called for each family with Canvas V.1.38^14^ using the ‘SmallPedigree-WGS’ workflow.^15^ SVs were called with Manta V.1.3.2.^16 17^ CNV/SV overlapping all genes in the EyeG2P data set (described in the Variant filtering section) were examined.

#### *De novo* analysis

For parent–child trios, short variants arising *de novo* in the child were identified using VASE^18^ with the following criteria: read depth ≥10 in parents and the child, genotype quality score ≥30 in the child and ≥20 in both parents, variant allele frequency ≥0.3 in the child and <0.05 in both parents, with a maximum of one variant allele called at site and variant allele absent from gnomAD V.3.0. Potential *de novo* variants were subsequently filtered to exclude low complexity, telomeric and centromeric regions, and to select only variants within coding regions, splice regions (exonic positions within 3 bp of an intron/exon junction or intronic positions within 8 bp of an intron/exon junction) or intronic variants with a SpliceAI^19^ delta score ≥0.5.

### Variant filtering

From all the variants identified in an individual, we selected only those that are rare, predicted to be functional and potentially relevant to eye disorders by using the G2P plugin^20^ ^21^ in VEP (V.90.1 (16)) and the Eye Gene Panel (https://www.ebi.ac.uk/gene2phenotype/downloads; accessed 29 August 2018). In short, we extracted only variants satisfying the inheritance requirements of the genes in the Eye Gene Panel, with minor allele frequency (MAF) in public databases <0.0001 for monoallelic and X-linked genes and MAF <0.005 for biallelic genes and annotated by VEP to have one of the following consequences: stop gained, stop lost, start lost, frameshift variant, inframe insertion/deletion, missense variant, coding sequence variant, initiator codon variant, transcript ablation, transcript amplification, protein altering variant, splice donor/acceptor variant (ie, canonical splice site) or splice region variant (ie, either within 1–3 bases of the exon or 3–8 bases of the intron).

### Detection of intronic splice variants

Variants within the *PAX6* locus (chr11:31784779-31817961; GRCh38) were annotated with SpliceAI delta scores using SpliceAI V.1.3^19^ using transcript coordinates from the ‘GENCODE basic’ transcript set from Ensembl V.95.

### *Cis*-regulatory variant analysis

The GRCh38 coordinates of 35 different CREs chosen for analysis are given in online supplemental table S4. A BED file was created from this table and BEDTools^22^ was then used to extract the *PAX6* CRE variants from the cohort VCF file. Subsequent filtering used the gnomAD^23^ allele frequency data in the VCF file.

Variant nomenclature (Human Genome Variation Society (HGVS)) was checked using Alamut software (Sophia Genetics) and VariantValidator.^24^ Variant numbering is according reference sequences NC_000011.10 (GRCh38), NM_000280.4 (-5a, 13 exons) and NP_000271.1 (422 amino acids).

### Experimental analysis of splice variants

#### RT-PCR and nested PCR of LCL-derived RNA

Patient-derived LCLs were recovered from liquid nitrogen storage. The cells were grown in suspension in Roswell Park Memorial Institute (RPMI) media containing 15% fetal calf serum (FCS) and penicillin/streptomycin, and incubated at 37°C/5% CO_2_. RNA was extracted from LCLs following the principles of the phenol-chloroform method^25^ using TRIzol reagent (Invitrogen) following the manufacturer’s instructions.

DNase treatment was performed using TURBO DNase kit (Invitrogen). cDNA was obtained from total RNA using the Super-Script First-Strand Synthesis System for RT-PCR kit (Invitrogen). A nested PCR across the *PAX6* locus was performed using four overlapping RT-PCR primer pairs spanning exons 1–5, exons 3–8, exons 7–12 and exons 9–13 (online supplemental table S2). The products were run on a 2% low melting point agarose gel with ethidium bromide. Bands of interest were excised and a gel DNA extraction was performed (Zymoclean Gel DNA Recovery Kit, Zymo Research, Freiburg im Breisgau) and sent for Sanger sequencing to look for mis-splicing.

## Results

### Assembling the cohort

DNA samples were available for 443 individuals with aniridia from 347 families recruited to the MRC Human Genetics Unit Eye Malformation Study. Forty-five families considered to be ‘*PAX6*-negative’ on previous screening were considered for inclusion in the WGS analysis. Of the 45 families, 3 were excluded on the basis of quality or quantity of the stored DNA and 4 were excluded following identification of various gene-disruptive variants via amplicon-based resequencing of all *PAX6* coding exons in all probands. Another family, RPID 1201, was excluded when a deletion of a critical *cis-*regulatory region 3′ of the *PAX6* gene (online supplemental figure S1) was found using droplet digital PCR across the *PAX6* locus following prescreening of 13 randomly chosen unrelated probands for CNVs.

DNA samples from a final cohort of 39 affected individuals from 37 families together with 12 unaffected relatives were sent for WGS. The family structures comprised 29 singleton probands, 6 trios (proband plus both unaffected parents) and 2 affected relative pairs (online supplemental figure S2). The proband phenotypes and molecular analyses of *PAX6* locus performed prior to this study (including the results from the referring centre) are detailed in table 1.

The following analyses of WGS data to identify sequence variants and SVs were performed in parallel.

### Identification of sequence variants in the *PAX6* transcriptional unit and regulatory region from WGS

WGS VCF files for all 51 individuals were filtered with the VEP-G2P plugin^21^ using the EyeG2P^20^ data set to detect high-impact and moderate-impact changes within genes known to cause genetic eye disease. Likely pathogenic *PAX6* sequence variants identified, described in the following, are listed in table 2 along with the American College of Medical Genetics and Genomics (ACMG) pathogenicity classifications.^1^ ^2^

This revealed one proband (RPID 2134) with a novel frame-shift variant (NM_000280.4 PAX6: c.842_843insGT) affecting a coding exon of *PAX6* constituting the sole ‘false negative’ for the prior screening of the *PAX6* coding region (WGS data viewed in IGV in online supplemental figure S3). This case had been screened many years previously using denaturing HPLC analysis. Although this was one of the ‘trios’, the ‘paternal’ sample was erroneously a duplicate of the maternal sample (short tandem repeat profiling in online supplemental table S3). We were thus unable to confirm whether the variant detected was *de novo*.

#### Essential splice site variants

**Figure 1:**
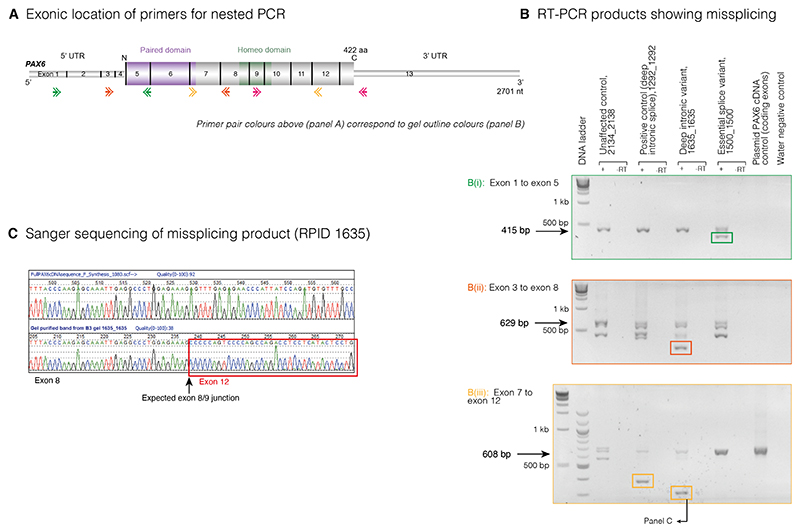
(A) Location of the four RT-PCR nested primer pairs spanning exons 1–5 (‘green’ pair), exons 3–8 (‘orange’ pair), exons 7–12 (‘yellow’ pair) and exons 9–13 (‘pink’ pair; products not shown as there was no evidence of mis-splicing in any of the cases). (B) Agarose gel images showing the RT-PCR products from the LCL-derived cDNA template. Samples are labelled as FID_RPID (family identifier_individual research participant identifier). (B(i)) Evidence of mis-splicing using green primer pair covering exons 1–5 in RPID 1500 (NM_000280.4:c.-128-2del). (B(ii)) The orange pair spanning exons 3–8 showed unexpected mis-splicing in RPID 1635 (intron 8 donor gain). (B(iii)) The yellow primers covering exons 7–12 showed mis-splicing in RPID 1635 and RPID 1292 (previously identified deep intronic variant in intron 8, not from this cohort and included as a positive control) suggestive of exon skipping. (C) Sanger sequence of gel-purified mis-spliced band confirming skipping of exons 9, 10 and 11 in RPID 1635. LCL, lymphoblastoid cell line.

Four families (three singletons, one trio) have heterozygous essential splice site (ESS) variants flanking exon 3, part of the *PAX6* 5′UTR. In RPID 877 and RPID 1019, both variants affect the 5′ base of intron 3 (IVS3+1, or c.-52+1), G>C and G>T, respectively (online supplemental figure S4A). Only the latter variant has been reported previously.^26^ A different variant at the same position (c.-52+1G>A) has been previously reported as resulting in skipping of exons 3, 4, 5 and 5a^27^; exon 4 contains the translation start site. RPID 1500 and RPID 5645 have identical and previously reported ESS variants (NM_000280.4 (*PAX6*): c.-128-2del (IVS2-2)).^28–30^ This variant occurred *de novo* in RPID 5645 (online supplemental figure S4B). The predicted effects of these sequence variants on splicing using the predictors in Alamut and SpliceAI are detailed in online supplemental table S4. Nested RT-PCR was performed on LCL-derived cDNA from RPID 1500 and showed evidence of abnormal splicing between exons 1 and 5 (figure 1B(i)).

#### Deep intronic variants affecting splicing

In RPID 3612, a variant was detected in intron 6 (NM_000280.4(-PAX6):c.357+334G>A) (online supplemental figure S5A). SpliceAI, SSF, MaxEnt and NNSPLICE all predict this to result in a donor gain (online supplemental table S4). One previous occurrence of this variant has been reported in aniridia.^29^ In RPID 1635, a novel *de novo* variant within intron 8 (NM_000280.4(-PAX6):c.682+68C>G) was identified (online supplemental figure S5B). SpliceAI, SSF, MaxEnt and NNSPLICE predict a donor gain consequence (online supplemental table S4). Nested RT-PCR was performed on LCL-derived cDNA from RPID 1635 and showed abnormal-sized bands using the primers spanning both exons 3–8 and 7–12. Sequencing the exons 3–8 product revealed unexpected skipping of exon 5 and part of exon 6. The exons 7–12 product-derived sequence showed skipping of exons 9, 10 and 11 (figure 1).

#### Variants in *PAX6* CREs

To identify causative CRE mutations at the *PAX6* locus, we first created a BED file listing the GRCh38 genome coordinates of 35 previously characterised CREs^31^ ^32^ (online supplemental table S5). Intersecting this BED file with the annotated cohort VCF file identified 39 variants that passed quality filters and were present in at least 1 out of 52 sequenced individuals (online supplemental table S6). Of 35 CREs examined, 24 encompassed one or more variant (online supplemental table S5). Of 39 variants, 20 had allele count within the study population of nine or greater. Of the remaining 19 variants, 15 had allele counts of one. Of 39 variants, 38 were present in gnomAD (online supplemental table S6), and the variant absent from gnomAD was a non-transmitted allele from an unaffected father in a trio.^23^ No *de novo* CRE SNVs or INDELs were identified. It thus seems very unlikely that any of the CRE variants are of clinical significance for aniridia.

#### Categorisation of *de novo* variants in genes other than *PAX6*

The only individual with *de novo* SNVs or INDELs outwith *PAX6* and with no causative SVs (described in the next section) was RPID 2469, who had five such variants (online supplemental table S7). This was the aforementioned trio for which the parents’ DNA was extracted from LCLs. Only the variant in the gene encoding adenosylhomocysteinase 3 (*AHCYL2*: p.(Leu352Met)) merited further consideration. This variant (NM_015328.4(AH-CYL2):c.1054C>A) is not present in gnomAD, and has CADD and REVEL scores of 24.3 and 0.73, respectively. There is no known Mendelian disease–gene link for *AHCYL2* and no claim can be made on the clinical significance of this variant.

### Identification of large-scale SVs from WGS

A combination of direct inspection of candidate loci using the IGV^33^ and genome-wide bioinformatic tools (Canvas^34^ and Manta^16^) was used to identify SVs from the available WGS data. A total of 17 different ultra-rare heterozygous SVs affecting *PAX6* and *FOXC1* were detected in 15 families (table 3 for genomic coordinates).

When compared with direct visual inspection, Canvas detected all deletions >10 kb at the *PAX6* and *FOXC1* loci (online supplemental figure S6) but not the two smallest *PAX6* deletions (126 bp, RPID 75, and 1.36 kb, RPID 1524). Canvas also called the *PAX6* deletion-duplication in RPID 1271 but was unable to detect the inversions in RPID 535 and RPID 774 as it uses read depth only. Manta detected all likely causative SVs but together with many false positive calls, so in practice these were identified solely by direct visualisation of the breakpoint regions using IGV.

#### Whole or partial deletions of *PAX6*

**Figure 2:**
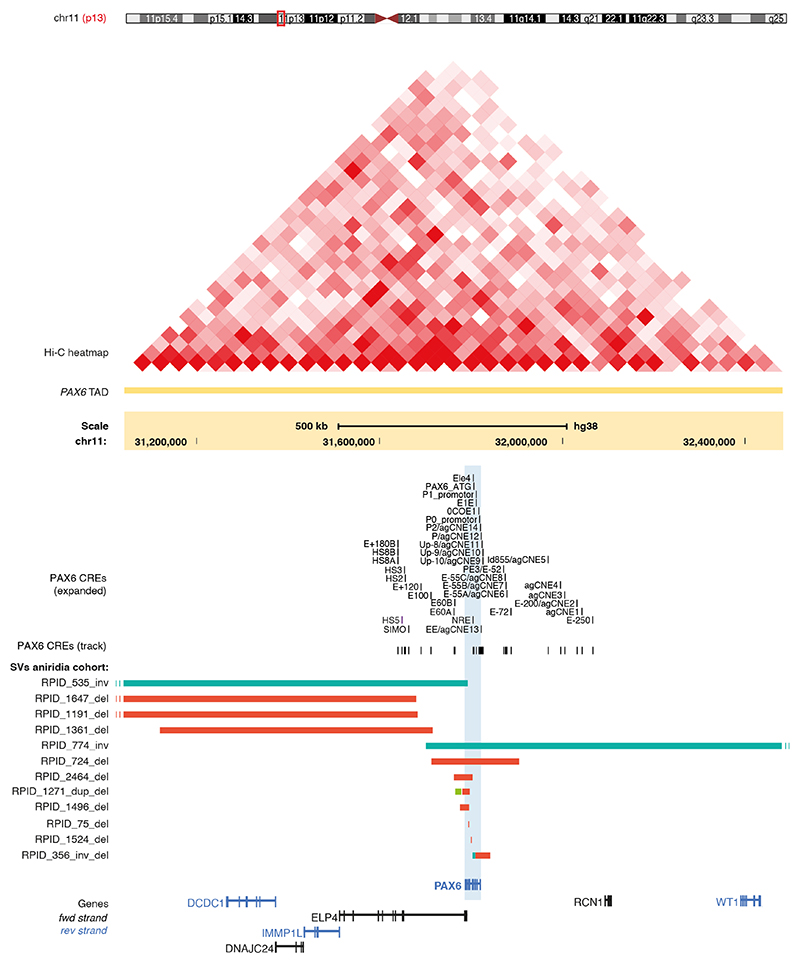
Structural variants (SV) identified on WGS affecting the wider *PAX6* locus. Each SV is shown as horizontal bars (inv, inversion, teal; del, deletion, red; dup, duplication, green). The *PAX6* topologically associated domain (TAD) is indicated by the Hi-C heatmap. The position of *PAX6 cis*-regulatory elements (CREs) is shown as track. Gene regulatory features such as the promoters and ATG are included. The position of *PAX6* is shaded blue. Seven of the SVs have intragenic breakpoints and RPID 724 has a whole gene deletion. Three SVs are deletions of the downstream regulatory region, taking out CREs implicated in aniridia, notably SIMO and HS5. Similarly, the SV seen in RPID 774 inverts *PAX6*, disrupting its relationship with these enhancers. A smaller scale version of this figure, showing the full span of the largest SVs, is shown in online supplemental figure S9. RPID, research participant identifier; WGS, whole genome sequencing.

Five individuals or families were found to have simple heterozygous deletions involving the *PAX6* transcription unit (figure 2, online supplemental figure S7): a whole gene deletion of 191 kb (RPID 724) and four partial deletions of 19 kb (RPID 1496), 1.4 kb (individual 1524), 0.13 kb (RPID 75) and 41 kb (RPID 2464). Each of these variants is expected to result in *PAX6* haplo-insufficiency. The variant detected in individual 1524 was subsequently found to have been identified independently by others.^35^

#### Deletions encompassing *PAX6 cis*-regulatory domains

RPID 1191, RPID 1361 and RPID 1647 were identified with likely causative deletions encompassing well-characterised CREs that control the developmental expression of *PAX6* (figure 2, online supplemental figures S8 and S9).

#### Balanced structural rearrangements disrupting *PAX6*

Two inversions of chromosome 11 were detected with break-points within or very close to *PAX6* (figure 2, online supplemental figures S9 and S10). The *PAX6* gene is directly disrupted in RPID 535, while in RPID 774 the breakpoint is between *PAX6* and the critical CREs SIMO and HS5.

#### Complex structural rearrangements disrupting *PAX6*

**Figure 3:**
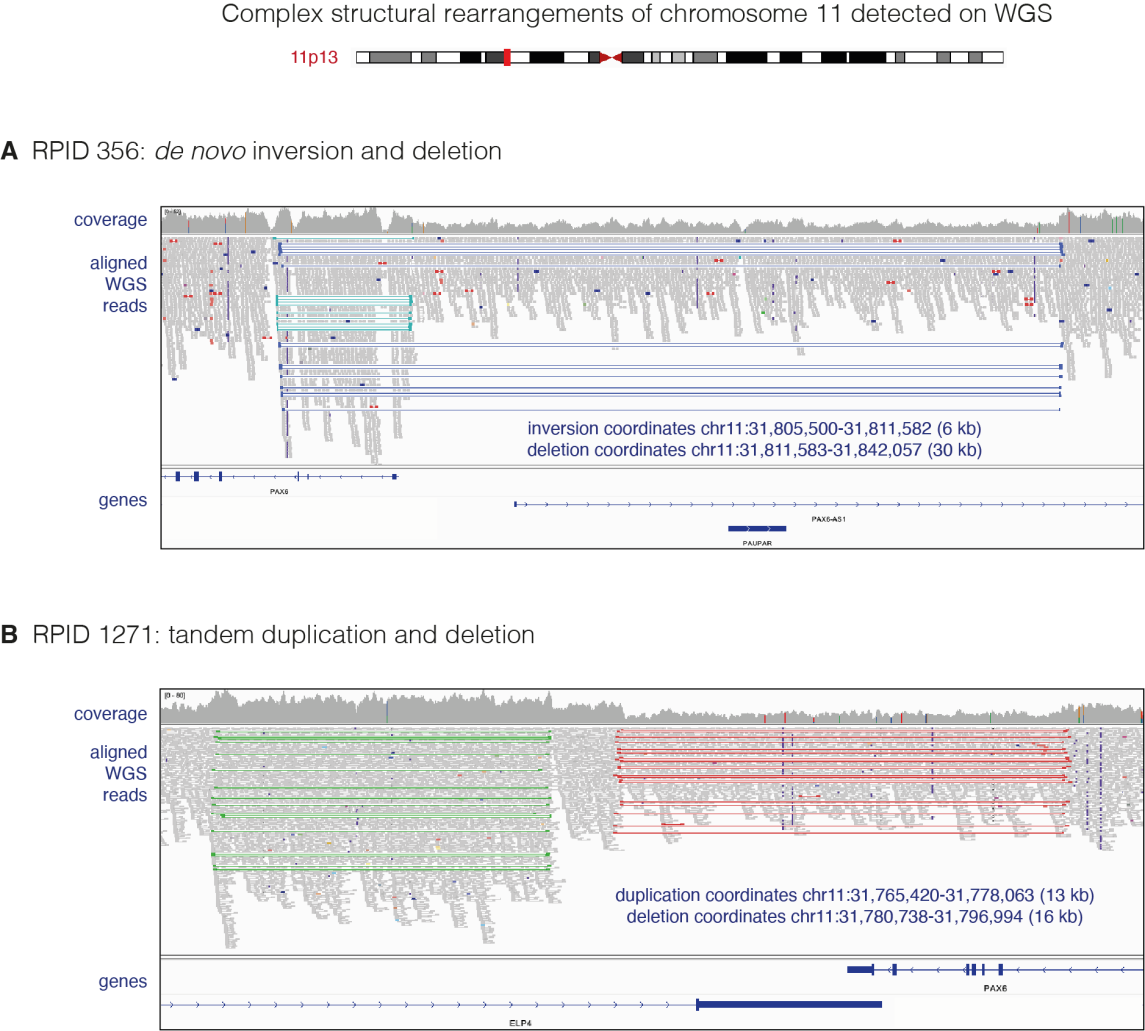
Complex structural rearrangements of chromosome 11 in two unrelated individuals with aniridia. Aligned WGS data viewed with IGV, with reads viewed as pairs and coloured both by insert size and by pair orientation. Coordinates estimated from WGS data (GRCh38). (A) RPID 356: an individual with sporadic bilateral aniridia and congenital cataracts. Trio WGS data, including the unaffected parents, are shown in online supplemental figure S11. WGS data show a *de novo* 6 kb inversion involving the P1 promoter, all of the 3’UTR and the first coding exon (exon 4) of *PAX6*; next to this is a 30 kb deletion, which deletes the P0 promoter and several enhancers, including EE. The blue and teal colours both denote paired reads with abnormal pair orientation. In IGV, pair orientation is determined first, and only if this is as expected will abnormal insert size then be flagged. Therefore, the reads across this deletion are not flagged in red (as they would be in a simple deletion) as they also span the inversion. A drop in coverage depth is seen in the deleted area. (B) RPID 1271: an individual (single proband) with bilateral aniridia and cataracts. WGS data indicate a 16 kb deletion (red) involving the six last exons of *PAX6* and a 13 kb tandem duplication (green) affecting the final intron of *ELP4*. Coverage depth is increased over the putative duplication and decreased over the putative deletion. IGV, Integrative Genomics Viewer; RPID, research participant identifier; WGS, whole genome sequencing.

RPID 356 was found to carry a *de novo* 6 kb heterozygous inversion with breakpoints at start of *PAX6* in intron 4, with an adjacent 30 kb region of *PAX6* deleted. In proband 1271, a 16 kb deletion encompassing the final six exons of *PAX6* was associated with a 13 kb tandem duplication immediately 3′ of *PAX6* (figure 3; also figure 2 and online supplemental figures S9 and S11).

#### Deletions encompassing *FOXC1*

Three heterozygous chromosome 6p deletions encompassing *FOXC1* were identified in three probands: RPID 1142, RPID 1451 and RPID 1732 (table 3, online supplemental figure S12). These ranged from 33 kb to 83 kb in size. All three probands had aniridia; two out of three had glaucoma (one confirmed as congenital) and two out of three had congenital aortic or aortic valve anomalies (table 1). The combination of aniridia with congenital glaucoma and aortic valvular disease would be consistent with previously reported *FOXC1* deletions.^36^

#### Breakpoint identification in a coincidental *de novo* reciprocal translocation t(1,9)

RPID 356 has a *de novo* reciprocal translocation t(1,9)(p36.1; q22), which was detected by routine cytogenetic analysis following the clinical diagnosis of aniridia. Given that no *PAX6* coding region mutation was identified on initial screening, the family was referred to our study to determine whether the break-point of this translocation could identify a novel locus or mechanism causing aniridia. However, as shown above, this individual has a second SV which disrupts *PAX6* and explains the phenotype. Using IGV, discrepant paired-end reads mapped a single breakpoint on chromosome 1 and two different breakpoints on chromosome 9, consistent with a paracentric inversion on chromosome 9 and a reciprocal translocation with chromosome 1 (online supplemental figure S13). No clinical impact is suspected for these three breakpoints.

## Discussion

In purely diagnostic terms, short-read WGS has significant advantages over short-read whole exome sequencing (WES). First, WGS allows reliable analysis of the whole transcription unit of each gene. This power is evidenced by our identification of previously cryptic causative variants in the 5′UTR and deep intronic regions of *PAX6* in 6 out of 22 (27.3%) diagnosed cases. The 5′UTR ESS variants perturb *PAX6* splicing; however, consequential changes to the length of the *PAX6* upstream ORF^37^ and/or disruption of VAX2 binding^38^ may also have mechanistic significance. More notably, we detected two deep intronic variants and tested the functional consequence of the novel one, predicted to result in an intron 8 donor site gain, using cDNA from an LCL derived from the proband. We could demonstrate the expected exon skipping 3′ to this variant, but we also found aberrant splice events 5′ to this intron, suggesting a more complex effect on splicing. While WGS may have better coverage of 5′UTRs than WES, it is particularly the deep intronic regions where it has a unique advantage.

A second advantage of WGS is more uniform per base coverage when compared with WES. This significantly improves our ability to detect disease-associated balanced structural variants (bSV) and CNVs. Our initial CNV screen was performed via direct inspection of the coverage depth change and unexpected pairing of end sequencing in proband BAM files using IGV.^33^ This proved to be the most diagnostically rewarding analysis undertaken in this study, yielding 15 of the 22 new diagnoses. Of these 15, 13 were CNVs (10 at *PAX6* locus and 3 encompassing *FOXC1*) and 2 were balanced SVs (bSVs) with *PAX6*-disruptive breakpoints, the latter not easily detectable by bioinformatic SV calling with Manta due to noise. The high CNV yield^26^ ^39^ ^40^ reflects both prescreening of the cohort for *PAX6* coding variants and the historic nature of some samples in our cohort, as these anomalies would almost certainly be detected by modern high-resolution, array-based methods of copy number assessments now used in clinical diagnostic laboratories throughout the world. On the contrary, the two bSVs would be unlikely to be detected on standard clinical testing other than WGS. The identification of an apparently coincidental *de novo* balanced reciprocal translocation in RPID 356 is interesting but has been observed in other developmental disorders in which a second intragenic SV is subsequently determined to be causative.^41^

The third, and possibly most exciting, advantage of WGS in the diagnostic investigation of classic aniridia is the ability to identify causative *cis*-regulatory variants affecting the developmental expression of *PAX6*. CNVs and bSVs encompassing CREs of *PAX6* but leaving the transcription unit intact have been recognised as resulting in functional haploinsufficiency for many years.^42^ ^43^ Predicting the consequence of SNVs within CRE remains challenging, and currently only one *de novo* plausibly causative CRE SNV in classic aniridia has been reported.^44^ We did not identify any additional CRE SNVs in this study, although we did identify four SVs affecting only the *PAX6* downstream regulatory region (three deletions and one bSV), leaving the gene itself intact. A similar PAX6 bSV is recently reported amongst a large, more diverse clinical diagnostic cohort^45^.

On the basis of the work from others^36^ ^45^ ^46^ and ourselves,^47^ the identification of *FOXC1* deletions is not surprising from a human genetics perspective. There is remarkably little information about developmental genetic interactions between these two genes, although it has been shown that *FOXC1* is a downstream direct target of *PAX6* in the developing iris and ciliary body.^48^

A fourth strength of WGS is that it permits a search for new candidate genes, and the mechanisms of inactivating known genes, in unexplained cases. We did not identify any likely causative variants at loci other than *PAX6* or *FOXC1*. A study with a larger number of trios would have greater power to detect new candidate loci.

We consider that the data presented here provide evidence that short-read WGS merits consideration as a primary investigation for classic aniridia. It certainly should be considered in cases with a normal array-based assessment of genome-wide copy number and *PAX6* coding region sequencing. We are mindful that WGS analysis is not currently capable of explaining all cases of aniridia, and there remain 15 out of 37 families in this study in whom we have still not identified a causative variant. One useful emerging diagnostic technology is long-read nanopore-based genome sequencing. This may be particularly useful in identifying bSV missed by the short-read technologies.^49–51^

## Supplementary Material

Supplementary Materials

## Figures and Tables

**Table 1:** Clinical features and prior molecular analyses of the WGS cohort

Individual (RPID)	Family (FID)		Inheritance		*PAX6* screen method	11p13 del analysis		Other genes screened*		ddPCR CNV PA*X*6		Phenotype
Singletons												
182	182		Sporadic		Direct	aCGH		No		Normal		Partial aniridia, atrophic iris, congenital cataracts, nystagmus, corneal endothelial degeneration.
224	224		Unknown		Direct	aCGH		No		Normal		Aniridia.
535	535		Sporadic		Direct	aCGH		No		No		Bilateral aniridia.
660	660		Unknown		Direct	aCGH		No		Normal		Aniridia, possible foveal hypoplasia.
724	723		Familial		DHPLC	Uncertain		No		No		Aniridia, type 2 diabetes.
774	774		Unknown		DHPLC	No		No		Normal		Bilateral aniridia, cataract, drusen at the macula and nasal to the optic disc.
877	877		Unknown		DHPLC	FISH		No		Normal		Bilateral aniridia, glaucoma, cataracts in young adulthood.
999	999		Unknown		DHPLC	No		No		Normal		Partial aniridia.
1019	1019		Sporadic		DHPLC	FISH		No		No		Aniridia.
1142	1142		Sporadic		DHPLC	Uncertain		No		No		Aniridia.
1191	1190		Familial		DHPLC	Uncertain		No		No		Partial aniridia, cataracts with surgery in late 20s.
1271	1271		Sporadic		DHPLC	aCGH		No		No		Bilateral aniridia, cataract extraction both eyes as teenager.
1304	1304		Familial		DHPLC	Uncertain		No		No		Aniridia.
1358	1358		Familial		DHPLC	Karyotype		No		No		Aniridia, congenital glaucoma, spherophakia, high myopia from birth, consanguinity, affected sibling, likely recessive or gonadal mosaic.
1361	1361		Familial		DHPLC	FISH		No		No		Aniridia.
1451	1451		Unknown		DHPLC	FISH		No		No		Aniridia, congenital glaucoma, bicuspid aortic valve with mild aortic stenosis.
1468	1468		Unknown		DHPLC	FISH		No		No		Bilateral aniridia.
1496	1496		Unknown		DHPLC	FISH		No		No		Aniridia.
1500	1500		Sporadic		DHPLC	aCGH		No		No		Bilateral aniridia, cataracts (posterior subcapsular), absent foveal reflexes, no nystagmus, vision right 6/12 N5 and left 6/36 N5, corneas clear with fine limbal vessels peripherally.
1524	1524		Familial		DHPLC	Unk		*CHX10*		No		Aniridia spectrum: iris and foveal hypoplasia, congenital corneal opacification with small lenses apposed to posterior surface of cornea.
1607	1607		Sporadic		DHPLC	FISH		No		No		Aniridia, congenital glaucoma.
1647	1646		Unknown		DHPLC	Unk		No		No		Aniridia with glaucoma.
1648	1648		Unknown		Direct	aCGH, FISH		No		No		Aniridia.
1732	1732		Unknown		DHPLC	Unk		No		No		Bilateral aniridia, glaucoma, opaque right cornea, aortic stenosis requiring neonatal surgery; not dysmorphic.
1879	1878		Unknown		DHPLC	FISH		No		No		Aniridia spectrum anterior chamber abnormality; multiple operations in early childhood for bilateral glaucoma.
1943	1943		Familial		DHPLC	‘11 p normal’		No		No		Bilateral aniridia.
2197	2197		Unknown		Unk; rescreened direct	‘chromosomes normal’		No		No		Bilateral aniridia, diaphragmatic eventration, undescended testicle and hydrocele; small kidneys.
3612	3612		Sporadic		Direct	aCGH, FISH		No		Normal		Bilateral aniridia, relatively well-preserved foveas, vision 0.4 logMAR† mild keratopathy as teenager, mild cataracts.
4340	4340		Sporadic		Direct	Unk		*SOX2, OTX2*		Normal		Bilateral aniridia, reasonably good vision.
Affected relative pairs												
1329	1326		Familial		DHPLC	Unk		No		Normal		Aniridia, nodular corneal dystrophy; relatives have classic aniridia.
1328					Not tested							Aniridia, nephew of 1326_1329.
2466	2464		Familial		DHPLC	Unk		No		No		Aniridia, half-sibling both affected.
2464					Not tested							Aniridia, half-sibling of 2464_2466.
Trio (affected child, unaffected parents)												
75	75		Sporadic		Direct	FISH, aCGH		No		Normal		Aniridia.
356	356		Sporadic		DHPLC	FISH		No		Normal		Bilateral aniridia, congenital cataracts, *de novo* 46,XX t(1;9)(p36.1;q22) found at amniocentesis.
1635	1635		Sporadic		DHPLC, plus direct for PD	aCGH		*FOXC1*, *SOX2*, *OTX2*		No		Variant aniridia: inferior iris defects, peripheral corneal vascularisation, inferior lens opacities, foveal hypoplasia, nystagmus, poor vision.
2134	2134		Sporadic		DHPLC	Likely FISH		No		No		Bilateral aniridia, nystagmus, glaucoma.
2469	2469		Sporadic		DHPLC	Unk		No		Normal		Aniridia, DNA from unaffected parents derived from lymphoblastoid cell lines.
5645	5645		Sporadic		Direct (by referring centre)	MLPA, microarray		*FOXC1*, *PITX2*, *ITPR1*		No		Bilateral partial aniridia.

‘Direct’ means direct Sanger sequencing.

* Other genes screened: results of any other relevant (non-*PAX6*) genes sequenced **prior** to inclusion in this study. Note *MAB21L1* was screened in all patients as part of the study.

† LogMAR is the logarithm of the minimum angle of resolution. aCGH, array comparative genomic hybridisation; ddPCR, droplet digital PCR (using four probes spanning PAX6/SIMO); del, deletion; DHPLC, denaturing high-performance liquid chromatography; FID, family identifier; MLPA, multiplex ligation-dependent probe amplification; PD, paired domain; RPID, research participant identifier; unk, unknown; WGS, whole genome sequencing.

**Table 2:** Likely pathogenic *PAX6* sequence variants (NM_000280.4)

Individual (RPID)	Family	Inheritance	Intron	GRCh38	CDS variant	Predicted consequence	ACMG/ ACGS	Previously reported
Coding loss-of-function variants								
2134	2134	Unknown	n/a	chr11:31793725_ 31793726insAC	c.842_843insGT	p.(Pro282Tyrfs*84)	P (0.999) PM2, PVS1, PP4 mod	No
Essential splice site variants affecting 5’ non-coding exons								
877	877	Unknown	IVS3+1	chr11:31806848C>G	c.-52+1G>C	p.(?) donor loss	LP (0.9) PM2, PS1 supp, PP3, PP4 mod*	No
1019	1019	Unknown	IVS3+1	chr11:31806848C>A	c.-52+1G>T	p.(?) donor loss	LP (0.949) PM2, PS4 supp, PP3, PS1 supp, PP4 mod*	Yes^26^
1500	1500	Unknown	IVS2-2	chr11:31806927del	c.-128-2del	p.(?) acceptor loss	LP (0.949) PM2, PS4 mod, PP3, PP4 mod	Yes^28–30 39^ VCV000430969.2
5645	5645	*De novo*	IVS2-2	chr11:31806927del	c.-128-2del	p.(?) acceptor loss	P (0.997) PM2, PS4 mod, PP3, PS2, PP4 mod	As above
Deep intronic donor gain variants								
1635	1635	*De novo*	IVS8+68	chr11:31794562G>C	c.682+68C>G	p.(?) donor gain	LP (0.988) PM2, PP3, PS2, PP4 mod	No
3612	3612	Unknown	IVS6+334	chr11:31801227C>T	c.357+334G>A	p.(?) donor gain	LP (0.9) PM2, PS4 supp, PP3, PP4 mod	Yes, as uncertain pathogenicity^29^ VCV000559621.2

ACMG/ACGS pathogenicity classification^1^ (and posterior probability calculated using the Bayesian framework tool in DECIPHER^2^).

* Arguable whether PVS1^1^ could be applied to this canonical splice site in a non-coding exon.

ACGS, Association for Clinical Genomic Science; ACMG, American College of Medical Genetics and Genomics; CDS, coding sequence; del, deletion; ins, insertion; LP, likely pathogenic; mod, moderate; n/a, not available; P, pathogenic; RPID, research participant identifier; supp, supporting.

**Table 3:** Likely pathogenic structural variants altering the *PAX6* or *FOXC1* loci

Individual (RPID)	Family	Variant type	Inheritance	Region affected†	Size (kb)	Genomic coordinates (GRCh38)
Simple deletions affecting *PAX6* transcription unit						
75	75	Deletion	*De novo*	*PAX6* intron 7/exon 8	0.126	chr11:31794763-31794889
724	723	Deletion	Unknown	*PAX6* whole gene deletion	191	chr11:31714074-31905175
1496	1496	Deletion	Unknown	C-termini of both *ELP4* and *PAX6* (intron 8 onwards)	19	chr11:31777022-31796016
1524	1524	Deletion	Unknown	*PAX6* exons 6 and 7	1.4	chr11:31800812-31802170*
2464	2464	Deletion	Segregating	C-termini of both *ELP4* and *PAX6* (intron 4 onwards)	41	chr11:31762967-31803741
Simple deletions altering *PAX6 cis*-regulation						
1191	1190	Deletion	Unknown	*PAX6* DRR	674	chr11:31009535-31683449
1361	1361	Deletion	Unknown	*PAX6* DRR	598	chr1 1:31 1 18961-31716799
1647	1646	Deletion	Unknown	*PAX6* DRR	842	chr11:30837881-31680038
Balanced rearrangements altering *PAX6* locus						
535	535	Inversion	Unknown	11p13-11p14.3 with intragenic *PAX6* breakpoint	7300	chr11:24479030-31792704
774	774	Inversion	Unknown	Inversion separating *PAX6*from DRR; breakpoints *ELP4* and *RAG2*	4900	chr11:31701464-36593500
Complex structural variants altering *PAX6* locus						
356	356	Inversion	*De novo*	N-terminus of *PAX6* up to intron 4	6	chr11:31805500-31811582
		Deletion		Upstream of *PAX6* (P0, EE enhancer)	30	chr11:31811583-31842057
1271	1271	Tandem duplication	Unknown	Final intron of *ELP4*	13	chr11:31765420-31778063
		Deletion		C-termini of both *ELP4* and *PAX6* (intron 8 onwards)	16	chr11:31780738-31796994
Simple deletions affecting *FOXC1*						
1142	1142	Deletion	Unknown	*FOXC1* whole gene deletion	82.6	chr6:1544064-1626702
1451	1451	Deletion	Unknown	*FOXC1* whole gene deletion	33.0	chr6:1581517-1615082
1732	1732	Deletion	Unknown	*FOXC1* whole gene deletion	81.6	chr6:1604452-1686063

* This individual’s structural variant was detected independently elsewhere and is patient 11 in the cited publication.^35^

† Exon numbering for PAX6 is as per NM_000280.4 (-5a, 13 exons)

DRR, Downstream regulatory region; RPID, research participant identifier.

## Data Availability

Data are available upon reasonable request. WGS sequence data have been deposited at the European Genome-Phenome Archive (EGA), which is hosted by the EBI and the CRG, under accession number EGAS00001006878. This was deposited in July 2023 and will soon be available publicly. GitHub links and publications for the various components are provided within the Methods section. Further codes are available from the corresponding author on reasonable request.

## What is Already Known on this Topic

⇒ Aniridia is a strikingly specific phenotype, carrying a 90%–95% positive predictive value for *PAX6* haploinsufficiency.

## What this Study Adds

⇒ This is the first dedicated whole genome sequencing (WGS) study of a classical aniridia cohort, highlighting the diagnostic power of WGS even in well-characterised Mendelian disorders.
⇒ It offers particular advantages in detecting deep intronic variants, *cis*-regulatory, and balanced or complex structural variants.

## How this Study Might Affect Research, Practice or Policy

⇒ This study suggests that short-read WGS merits consideration as a primary investigation for classic aniridia.
