# Supplementary material for: Short-read whole genome sequencing identifies causative variants in most individuals with previously unexplained aniridia
Source: J Med Genet. Author manuscript; Available in PMC 2024 Jun 25. (PMC7615962; doi:10.1136/jmg-2023-109181)
Supplement: Supplementary Materials [file EMS194103-supplement-Supplementary_Materials.pdf]

Supplementary Information & Figures

for Hall *et al*, Short-read whole genome sequencing identifies causative variants in most individuals with previously unexplained aniridia

Contents

Supplementary Materials and Methods

Supplementary Figures

Hall HN, *et al. J Med Genet* 2023;0:1–12. doi: 10.1136/jmg-2023-109181

Supplementary Materials and Methods

Droplet digital PCR

Droplet digital PCR with 4 amplicons spanning the wider *PAX6* locus was performed as a pilot screen in 13 individuals prior to finalising the WGS cohort, to identify copy number variants (CNVs) which had not been adequately assessed prior to this study. Primers (Universal Probe Library assay design centre, Roche) and UPL probe pairs were targeted to three intragenic *PAX6* regions (exon 2, intron 7, 3'UTR) and the critical SIMO enhancer in the downstream regulatory region [Link to primer table]. PCR reaction mixtures were prepared (20 µl reaction containing 2 µl gDNA at 20 ng/µl, 10 µl of 2x ddPCR Supermix (Bio-Rad, Watford, UK), 2 µl of 2.5 µM UPL probe, 2 µl each forward and reverse primer (10 µM), 1 µl 20x RNaseP and 1 µl dH2O). The QX200 Droplet Digital PCR system (Bio-Rad) was used following manufacturer's protocols (droplet generation, PCR, and plate reader). Droplets were generated in the QX200 Droplet Generator; PCR was then performed under the following conditions: 95° C 10 min, (94° C 30 sec, 55° C 1 min) x40, 95° C 10 min. The plate was then analysed by the droplet reader and CNV count was analysed using Bio-Rad QuantaSoft software.

Whole genome sequencing (detailed methods)

WGS was performed on Illumina HiSeqX, by BGI (New Territories, Hong Kong) for 9 samples, and Edinburgh Genomics (Edinburgh, UK) for the remaining 42 samples.

For BGI Hong Kong:

Next generation sequencing libraries were prepared as follows:

DNA fragmentation

1µg genomic DNA was sheared by a Covaris ultrasonicator. The DNA fragments were assessed by gel electrophoresis, purified using AxyPrep Mag PCR clean-up kit (Axygen), incubated with End Repair Mix at 20°C for 30 min and then purified again. The repaired DNA fragments were A-tailed at the 3' end (37°C for 30 min), adapter ligated (16°C for 16 hr), purified and then size selected (350 bp) prior to PCR amplification and purification. MGIEasy DNA library construction kits (MGI Tech) were used.

For Edinburgh Genomics:

In addition to the QC outlined in the main manuscript, gDNA samples were further evaluated for quantity using Quant-iT Picogreen reagent (Thermo Fisher) and Spectramax XPS Gemini plate reader (Molecular Devices, Wokingham, UK), and quality using an AATI Fragment Analyzer (Agilent, Stockport, UK) (total amount >1000ng and quality score >5 passed sample QC). gDNA samples were pre-normalised to a concentration range of 5-40ng/uL.

Next generation sequencing libraries were prepared using an Illumina SeqLab specific TruSeq Nano High Throughput library preparation kit in conjunction with the Hamilton MicroLab STAR and Clarity LIMS X (4.2) Edition. 200ng gDNA sample inputs were sheared to a 450bp mean insert size using a Covaris LE220 focused-ultrasonicator. The inserts were blunt ended, A-tailed, size selected, with TruSeq adapters ligated to each fragment before PCR amplification.

Insert size for each library was evaluated using the Caliper GX Touch with a HT DNA 1k/12K/HI SENS LabChip and HT DNA HI SENS Reagent Kit, ensuring mean fragment sizes of 530bp to 950bp. The Roche LightCycler 480 and a Kapa Illumina Library Quantification kit were used to ensure the concentration of each library was between 5.5nM and 40nM. Libraries are normalised to 1.5nM and denatured for clustering and

sequencing at 300pM using a Hamilton MicroLab STAR with Genologics Clarity LIMS X (4.2) Edition. Libraries were clustered onto a HiSeqX Flow cell v2.5 on cBot2s and the clustered flow cell transferred to a HiSeqX for sequencing using a HiSeqX Ten Reagent kit v2.5.

Supplementary Figures

Supplementary Figure S1: Copy number variation (CNV) at the *PAX6* locus assessed by droplet digital (dd)PCR, revealing a heterozygous deletion involving the *cis*-regulatory element, SIMO. (A) The CNV of 4 probes spanning the *PAX6* locus were tested, 3 amplicons within the *PAX6* gene in turquoise (5' UTR in exon 2, the middle of the gene in intron 7, and 3' UTR in exon 13) and the final one, red, covering SIMO, a critical *cis*-regulatory enhancer region which is approximately 150 kb 3' to the start of *PAX6*. (B) FID/RPID 1201: the intragenic amplicons showed a normal copy number, but the 75 bp amplicon targeting SIMO showed a copy number of 1, indicating a heterozygous deletion and explaining the aniridia phenotype (1). Methods for this experiment are above in Supplementary Materials and Methods

A ddPCR amplicons

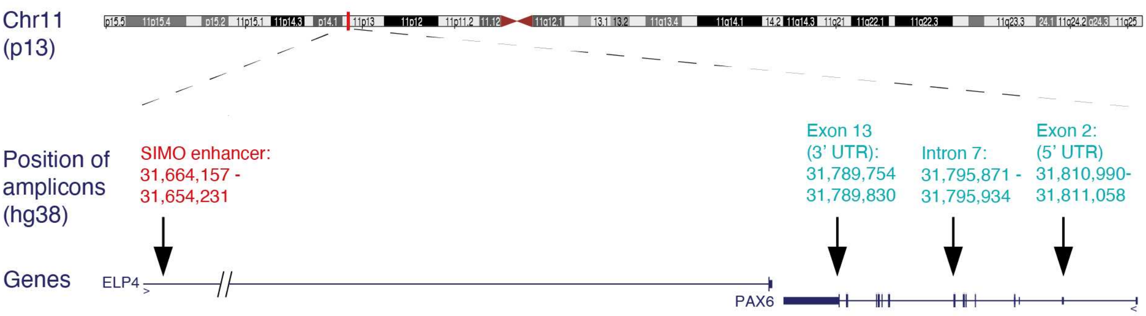

B Copy number determined by ddPCR, RPID 1201

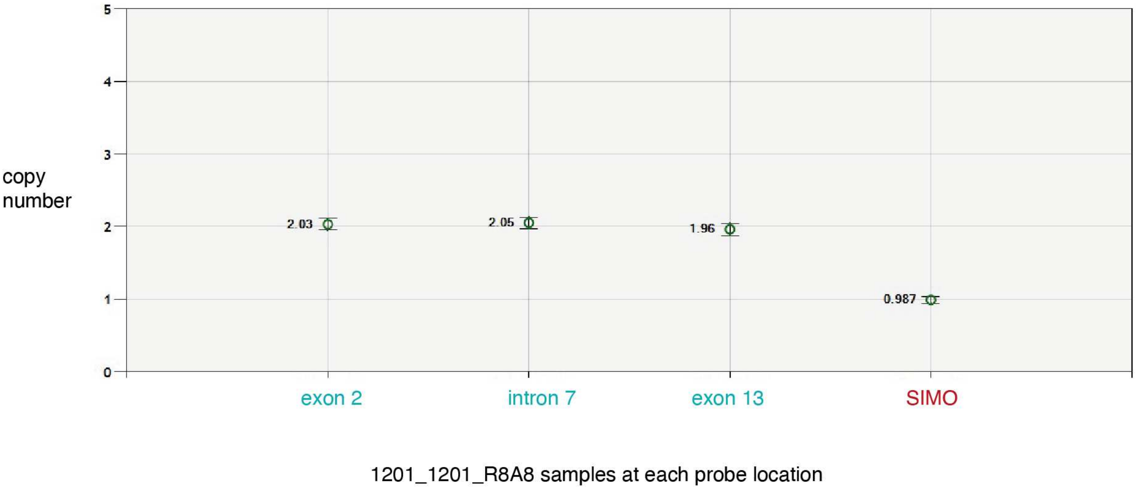

Hall HN, *et al. J Med Genet* 2023;0:1–12. doi: 10.1136/jmg-2023-109181

Supplementary Figure S2: Pedigrees for the two families included in the WGS cohort as duos (proband plus affected relative). Family members with aniridia are shaded in black. Those with an ID number underneath (FID_PID, family_personal ID respectively) were sent for WGS. (A) Family 2464, affected half siblings with aniridia sharing an unaffected father (I:2), who is presumed to have gonadosomatic mosaicism. The mother of 2464_2466 (I:1) is recorded as "?" as it is not known for certain that she was unaffected. (B) Family 1326, a large family with several members affected by aniridia. The aunt (proband; 1326_1329, II:1) and nephew (1326_1328, III:6) duo was included in the WGS cohort

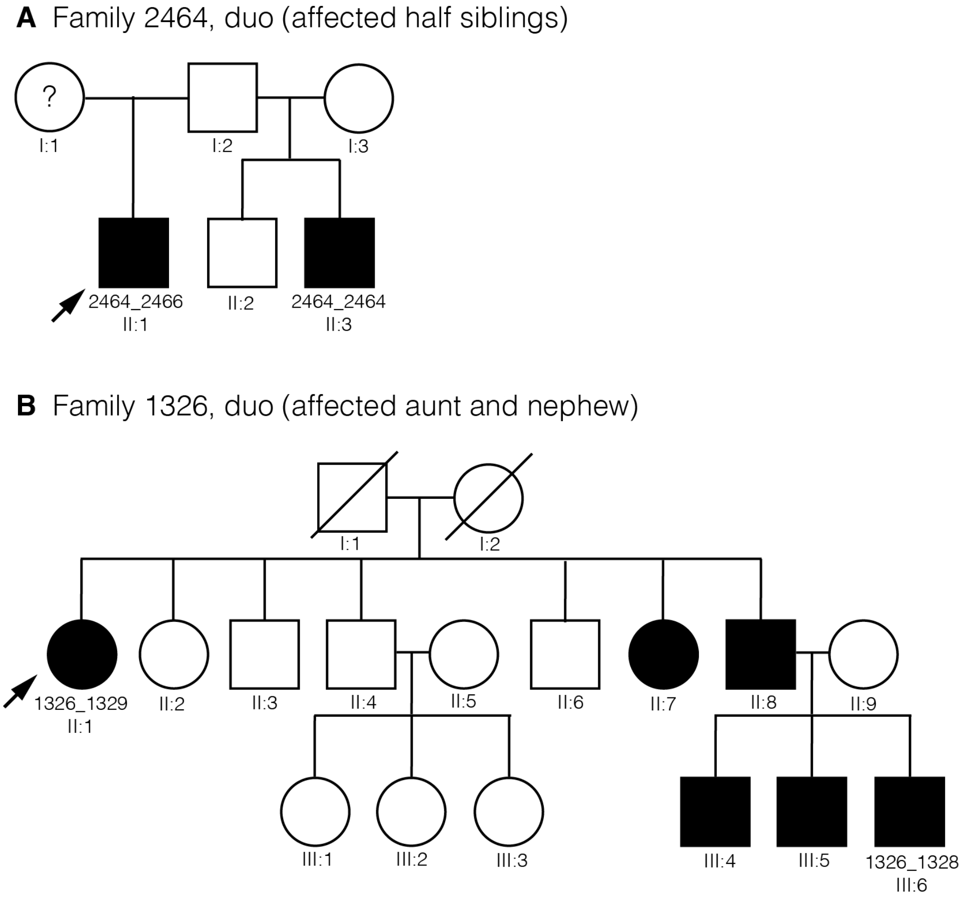

Hall HN, *et al. J Med Genet* 2023;0:1–12. doi: 10.1136/jmg-2023-109181

Supplementary Figure S3: Aligned WGS data for family 2134 showing a likely gene-disruptive sequence variant affecting exon 10 of *PAX6* in the proband (RPID 2134). The proband has sporadic bilateral aniridia, nystagmus and glaucoma. The IGV snapshot shows aligned WGS data from proband (top panel) demonstrating a 2 bp insertion (arrow) in exon 10 of *PAX6* (at chr11:31,793,725, NM_000280.4:c.842_843insGT), and below that of the unaffected mother not carrying the variant.

Sequence variant detected on WGS: 2 bp insertion *PAX6* exon 10 causing frameshift stop

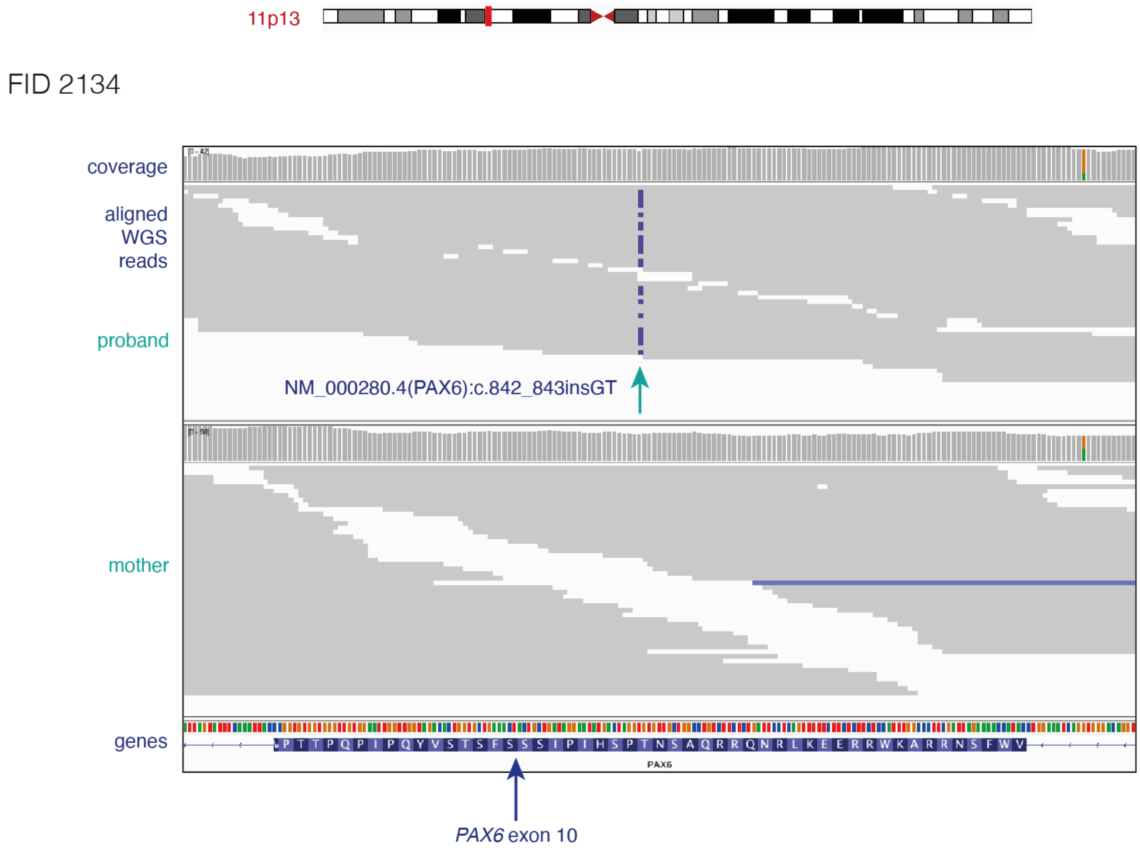

Hall HN, *et al. J Med Genet* 2023;0:1–12. doi: 10.1136/jmg-2023-109181

Supplementary Figure S4: Single nucleotide variants affecting essential splice sites of *PAX6*. Aligned WGS data (shown in IGV) showing 3 heterozygous variants (green arrows) in 4 families disrupting the essential splice sites flanking exon 3. (A) Single probands: RPID 877, who has bilateral aniridia, glaucoma and bilateral cataracts in young adulthood, has the heterozygous substitution Chr11(GRCh38):g.31806848C>G, NM_000280.4(PAX6):c.-52+1G>C (IVS3+1). This is visible at the marked arrow as a blue and orange bar on the coverage track. RPID 1019, an individual with sporadic aniridia, has a different substitution of the same nucleotide Chr11(GRCh38):g.31806848C>A, which is NM_000280.4(PAX6):c.-52+1G>T (IVS3+1), visible at the marked arrow as a blue and green bar on the coverage track. RPID 1500 has isolated bilateral aniridia, posterior subcapsular cataracts, absent foveal reflexes, subtle peripheral corneal changes and relatively good vision (6/12 N5 right, 6/36 N5); they were found to have Chr11(GRCh38):g.31806927del, which is NM_000280.4(PAX6):c.-128-2del (IVS2-2), a single base pair deletion affected the -2 position of intron 2. (B) Family 5645, a trio of child affected by bilateral aniridia and unaffected parents, carries exactly the same variant as RPID 1500. Please note splicing predictors are listed in **Table S4**.

Single nucleotide variants detected on WGS involving *PAX6* 5' UTR essential splice sites

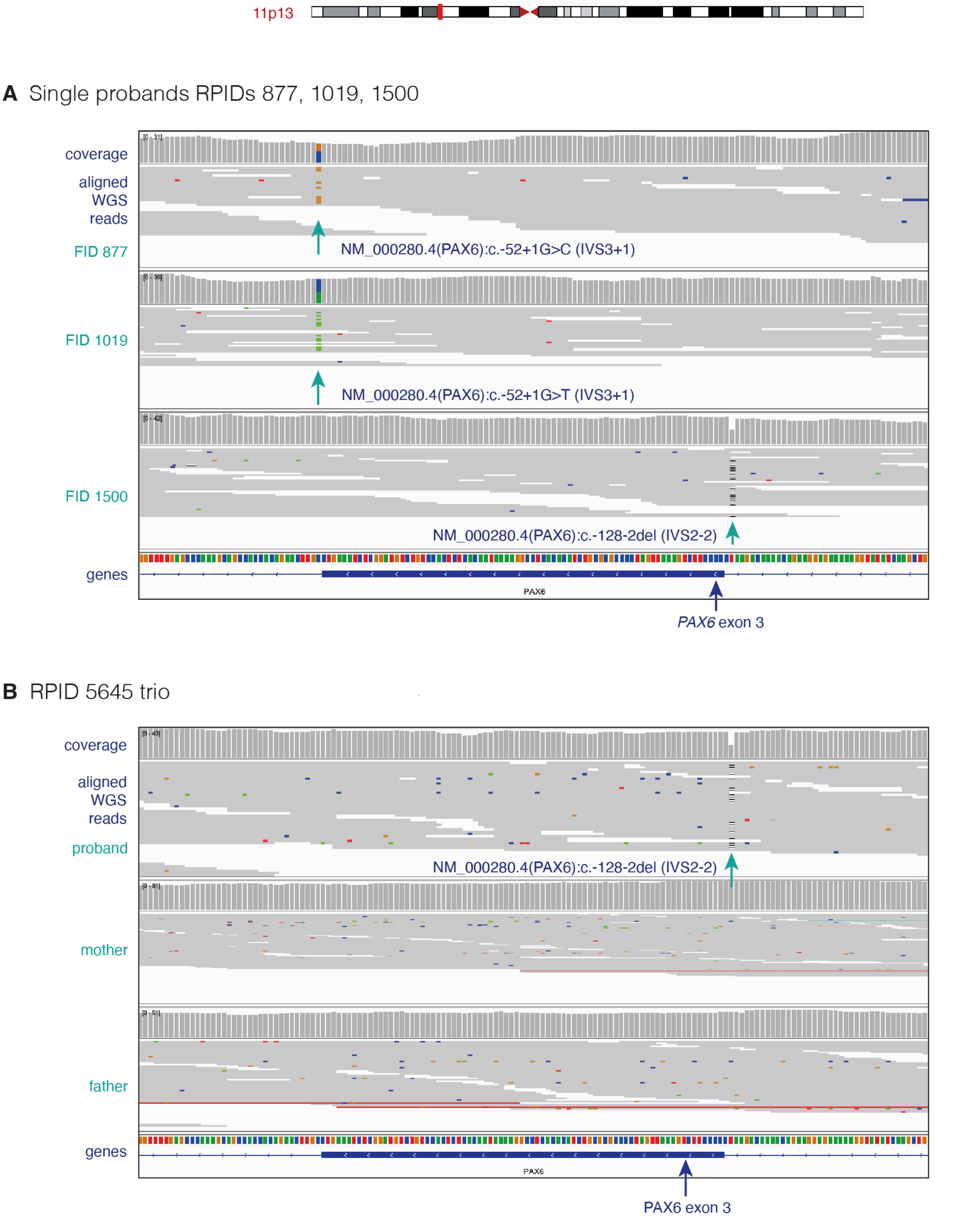

Supplementary Figure S5: Deep intronic splice variants detected on WGS. (A) RPID 3612 was found to have Chr11(GRCh38):g.31801227C>T, NM_000280.4(PAX6):c.357+334G>A, a deep intronic variant in intron 6. A 1 kb span of aligned WGS data is shown; (B) RPID 1635 was found to carry Chr11(GRCh38):g.31794562G>C, NM_000280.4(PAX6):c.682+68C>G *de novo*. The unanimous prediction of a donor gain from the Alamut splice predictors was consistent with the 0.97 confidence donor gain prediction of SpliceAI (**Table S4**). Experimental confirmation of missplicing for RPID 1635 is described in the main manuscript.

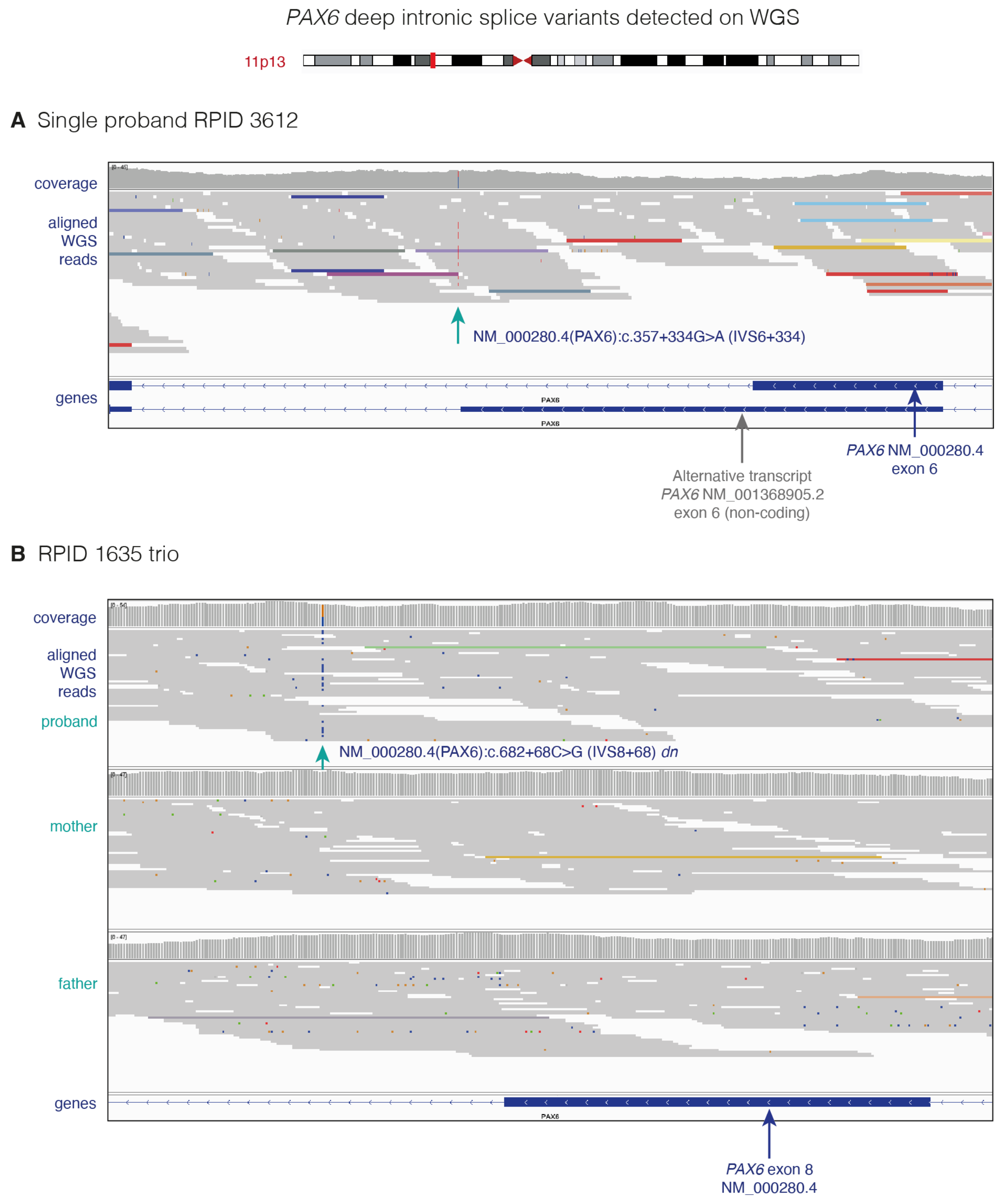
PAX6 deep intronic splice variants detected on WGS
11p13
A Single proband RPID 3612
coverage
aligned WGS reads
NM_000280.4(PAX6):c.357+334G>A (IVS6+334)
genes
PAX6
PAX6
PAX6 NM_000280.4 exon 6
Alternative transcript *PAX6* NM_001368905.2 exon 6 (non-coding)
B RPID 1635 trio
coverage
aligned WGS reads
proband
NM_000280.4(PAX6):c.682+68C>G (IVS8+68) *dn*
mother
father
genes
PAX6
PAX6 exon 8 NM_000280.4

Supplementary Figure 6: Bioinformatic CNV calling with Canvas across the (A) *PAX6* chromosome 11 and (B) *FOXC1* (chromosome 6) loci. These samplots figures show intersection of Canvas CNV calls for these two loci. Chromosomal coordinates (hg38) are at the bottom. Samples are labelled as FID_RPID (family ID_individual research participant ID). Annotations are shown from two databases of structural variation: Database of Genomic Variants (DGV), containing variants seen in healthy individuals, with blue (upper DGV track) showing copy number gain and red (lower track) showing CN loss; and dbVar (specifically, the nstd186, or NCBI Curated Common SVs study), which includes data from gnomAD Structural Variants, 1000 Genomes SV, and DECIPHER Common CNVs (2). (A) The trio FID 356, duo FID 2464 and the single probands (RPIDs 1496, 1271, 724, 1647, 1361 and 1191) all show reduced copy number (CN) at the *PAX6* locus consistent with a deletion, and 1271 additionally has an adjacent area of increased CN, consistent with duplication. (B) Three individuals with reduced CN over the *FOXC1* locus (RPID 1142, 1451, 1732).

Structural variant calling of WGS data with Canvas

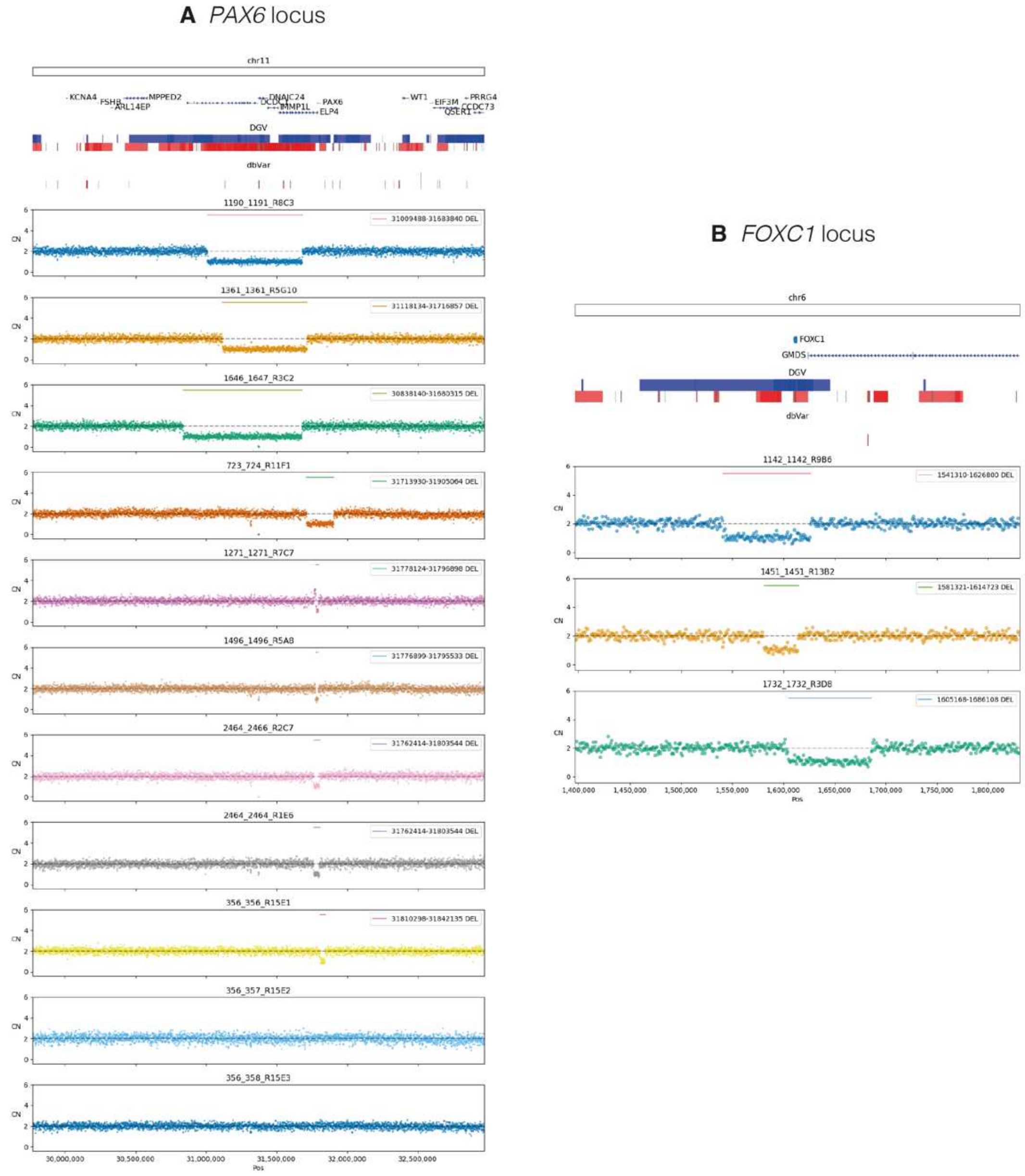

Hall HN, et al. J Med Genet 2023;0:1–12. doi: 10.1136/jmg-2023-109181

Supplementary Figure S7: SVs disrupting the PAX6 locus. IGV snapshot of the aligned WGS data are shown for probands identified by research participant ID number (RPID), and/or family ID. In the deletion cases a drop in coverage is evident in the track above the WGS reads. Genomic coordinates of the SV are given (hg38). The individual 150 bp reads are paired (viewed here with a line connecting each pair) and coloured by insert size, with a red colour suggesting a deletion. The equivalent colour coding for an insertion is dark blue for paired reads, or purple for a single base insertion within a read. noting SNVs are visible on the coverage track. (A) RPID 724: whole gene deletion of *PAX6* chr11:31,714,074-31,905,175. (B) RPID 1496: 19 kb deletion involving C-terminal portions of both *PAX6* and *ELP4* (tail-to-tail orientation) chr11:31,777,022-31,796,016. (C) RPID 1524: 1358 bp deletion involving exons 6 and 7 of *PAX6*, chr11:31,800,812-31,802,170. (D) FID 75 trio (proband, RPID 75): *de novo* 126 bp deletion involving the intron 7 and exon 8 of *PAX6*, chr11:31,794,763-31,794,889. (E) FID 2464 duo (RPID 2466, proband; RPID 2464, half brother): two affected half siblings with a 41 kb del

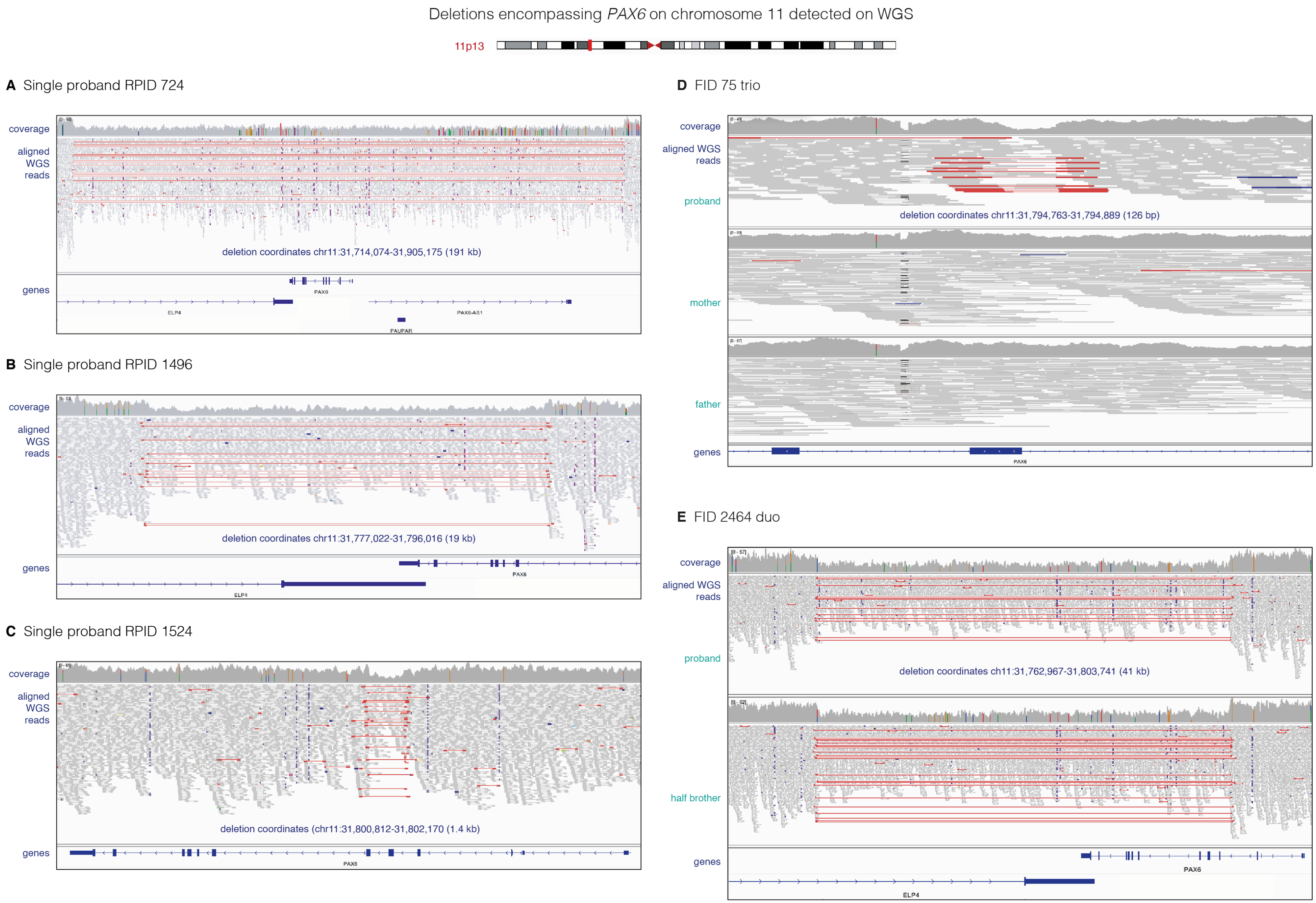
Deletions encompassing PAX6 on chromosome 11 detected on WGS
11p13
A Single proband RPID 724
coverage
aligned WGS reads
deletion coordinates chr11:31,714,074-31,905,175 (191 kb)
genes
PAX6
ELP4
PAX6-AS1
PAUPAR
B Single proband RPID 1496
coverage
aligned WGS reads
deletion coordinates chr11:31,777,022-31,796,016 (19 kb)
genes
PAX6
ELP4
C Single proband RPID 1524
coverage
aligned WGS reads
deletion coordinates (chr11:31,800,812-31,802,170 (1.4 kb)
genes
PAX6
D FID 75 trio
coverage
aligned WGS reads
proband
deletion coordinates chr11:31,794,763-31,794,889 (126 bp)
mother
father
genes
PAX6
E FID 2464 duo
coverage
aligned WGS reads
proband
deletion coordinates ch11:31,762,967-31,803,741 (41 kb)
half brother
genes
PAX6
ELP4

Supplementary Figure 8: Deletions detected on WGS involving the *PAX6* downstream regulatory region on chromosome 11. Aligned WGS data is shown for (A) RPID 1191: chr11:31,009,535-31,683,449; (B) RPID 1361: chr11:31,118,961-31,716,799; (C) RPID 1647: chr11:30,837,881-31,680,038.

Deletions on chromosome 11 involving the downstream regulatory region of *PAX6*

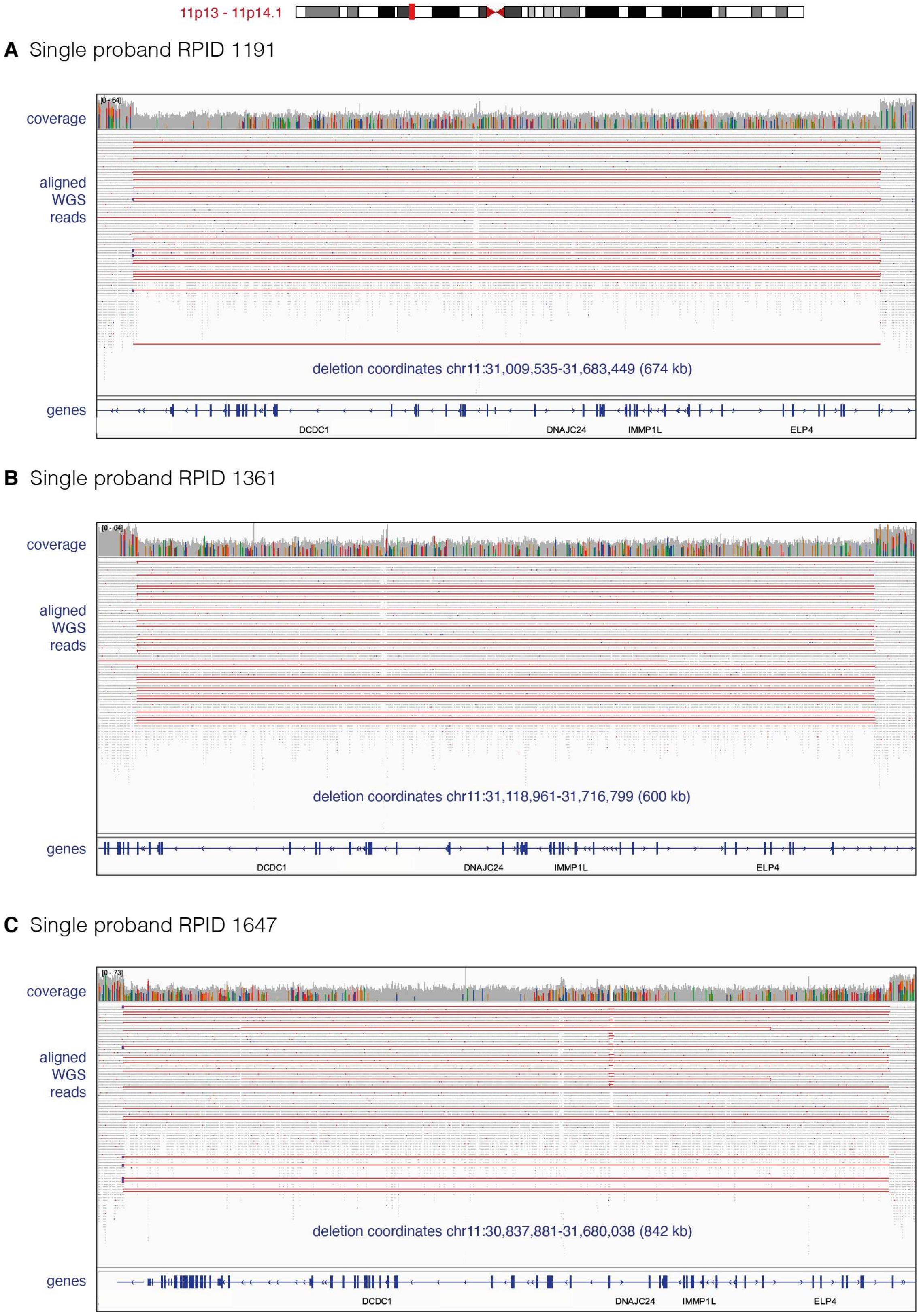

Supplementary Figure 9: Structural variants (SV) identified on WGS, their location on chromosome 11 and their relationship to *PAX6*. This figure shows approximately 12 Mb (just under one tenth) of chromosome 11, marked on the ideogram at the top and then expanded below. SVs are shown as horizontal bars (inv, inversion, teal; del, deletion, red; dup, duplication, green). The two largest SVs are RPID 535, 7.3 Mb, and RPID 774, 4.9 Mb, both inversions. The topologically associated domains (TADs) (3), illustrated by the Hi-C heatmap (data from cortex (4)) are marked in yellow and blue blocks. The area of the PAX6 TAD is marked off: this allows easy comparison with Figure 2 which shows this area in smaller scale (10x higher resolution) and allows us to see the relationship of the SVs with the *PAX6* cis-regulatory elements and neighbouring genes.

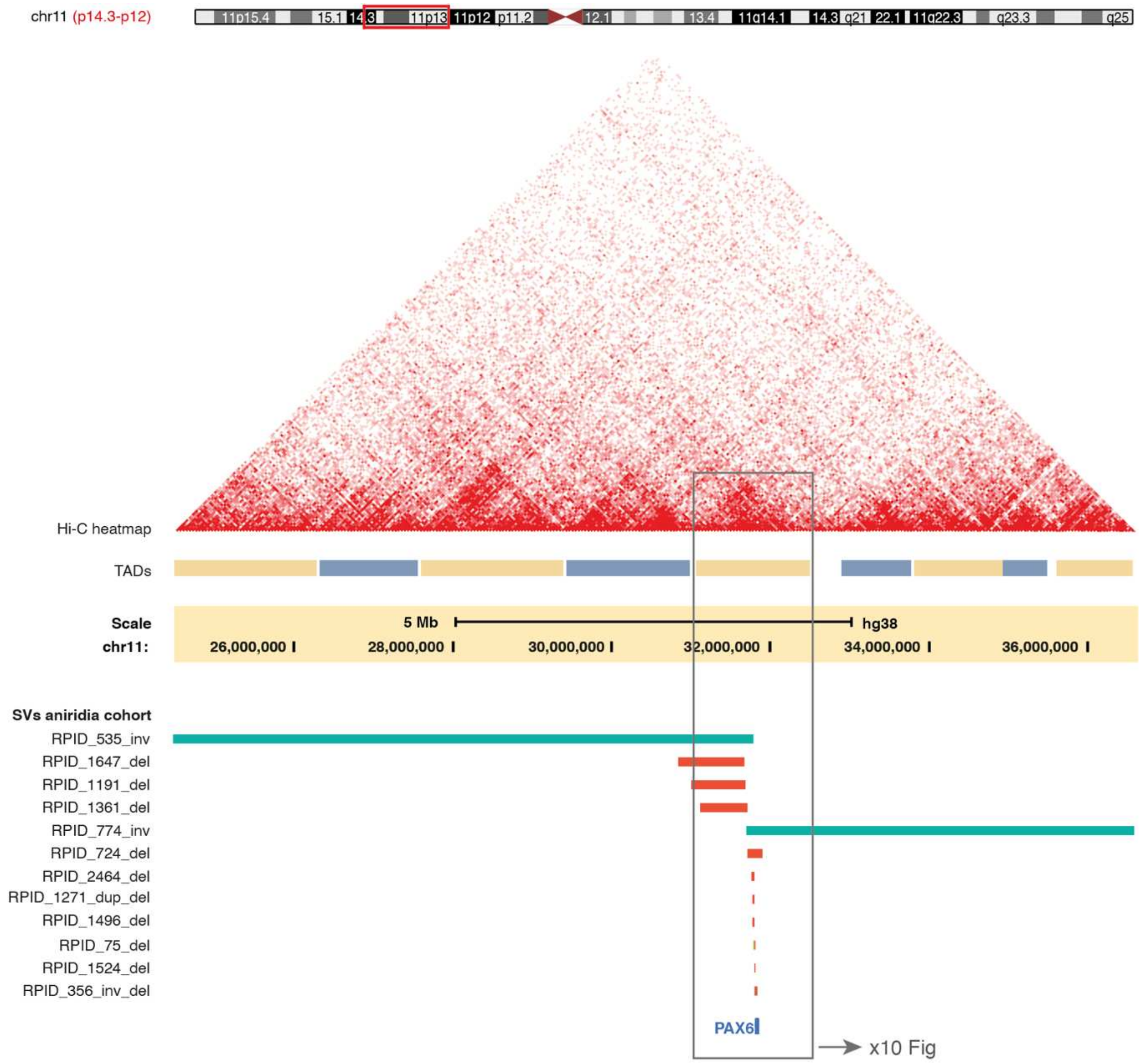

Hall HN, *et al. J Med Genet* 2023;0:1–12. doi: 10.1136/jmg-2023-109181

Supplementary Figure 10: The balanced inversions affecting the PAX6 locus were too large to capture in a single IGV view; a split-screen view showing both breakpoints is shown. The red box on the ideograms indicates the inverted region. Reads are coloured by pair orientation, with the blue and teal colours indicate that paired read has been found in an unexpected orientation to its mate (with respected to the reference genome) and implying an inversion. Consistent with the balanced nature of the rearrangement, no drop in coverage is seen on the coverage track. (A) RPID 535: a 7.3 Mb inversion of chromosome 11 and an intragenic *PAX6* breakpoint in intron 11; chr11:24,479,030-31792704. (B) RPID 774: An inversion of 4.9 Mb, which leaves *PAX6* intact but disrupts its relationship with critical enhancers; chr11:31,701,464-36,593,500.

Hall HN, *et al. J Med Genet* 2023;0:1–12. doi: 10.1136/jmg-2023-109181

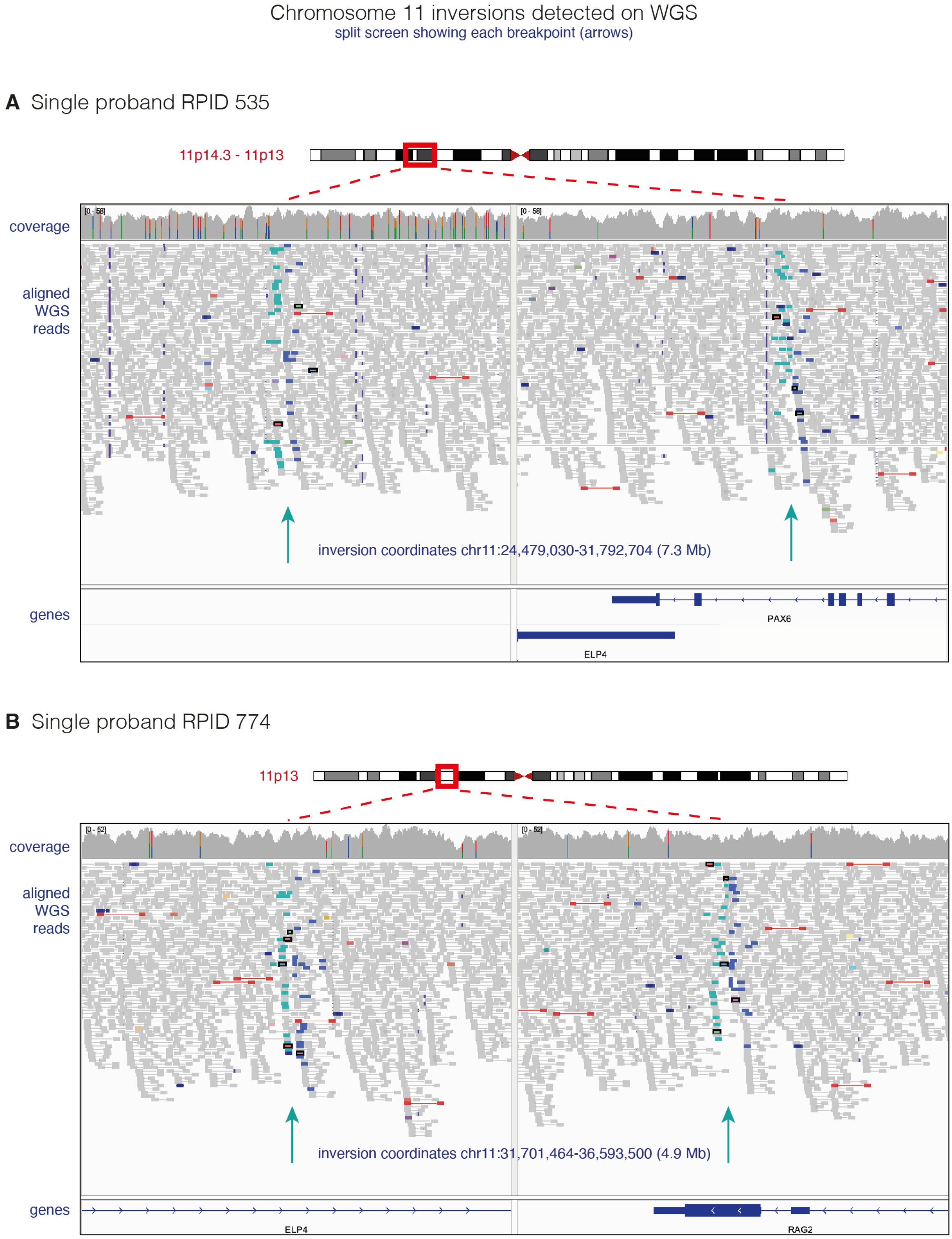
Chromosome 11 inversions detected on WGS
split screen showing each breakpoint (arrows)
A Single proband RPID 535
11p14.3 - 11p13
coverage
aligned WGS reads
genes
[0 - 58]
[0 - 58]
inversion coordinates chr11:24,479,030-31,792,704 (7.3 Mb)
PAX6
ELP4
B Single proband RPID 774
11p13
coverage
aligned WGS reads
genes
[0 - 52]
[0 - 52]
inversion coordinates chr11:31,701,464-36,593,500 (4.9 Mb)
ELP4
RAG2

Supplementary Figure 11: Complex structural rearrangements of chromosome 11 detected on WGS. (A) FID 356 trio (proband is RPID 356): a 6 kb inversion (chr11:31,805,500-31,811,582, teal) involving the P1 promotor, all of the 3'UTR and the first coding exon (exon 4) of *PAX6*; next to this a 30 kb deletion (chr11:31,811,583-31,842,057), which deletes the P0 promoter. A drop in coverage depth is seen in the deleted area. (B) RPID 1271: a 16 kb deletion involving the 6 last exons of *PAX6* (chr11:31,780,738-31,796,994, red) and a 13 kb tandem duplication (chr11:31,765,420-31,778,063, green) affecting the final intron of *ELP4*.

Complex structural rearrangements of chromosome 11 detected on WGS

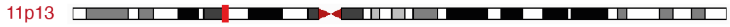

A FID 356 trio: *de novo* inversion and deletion

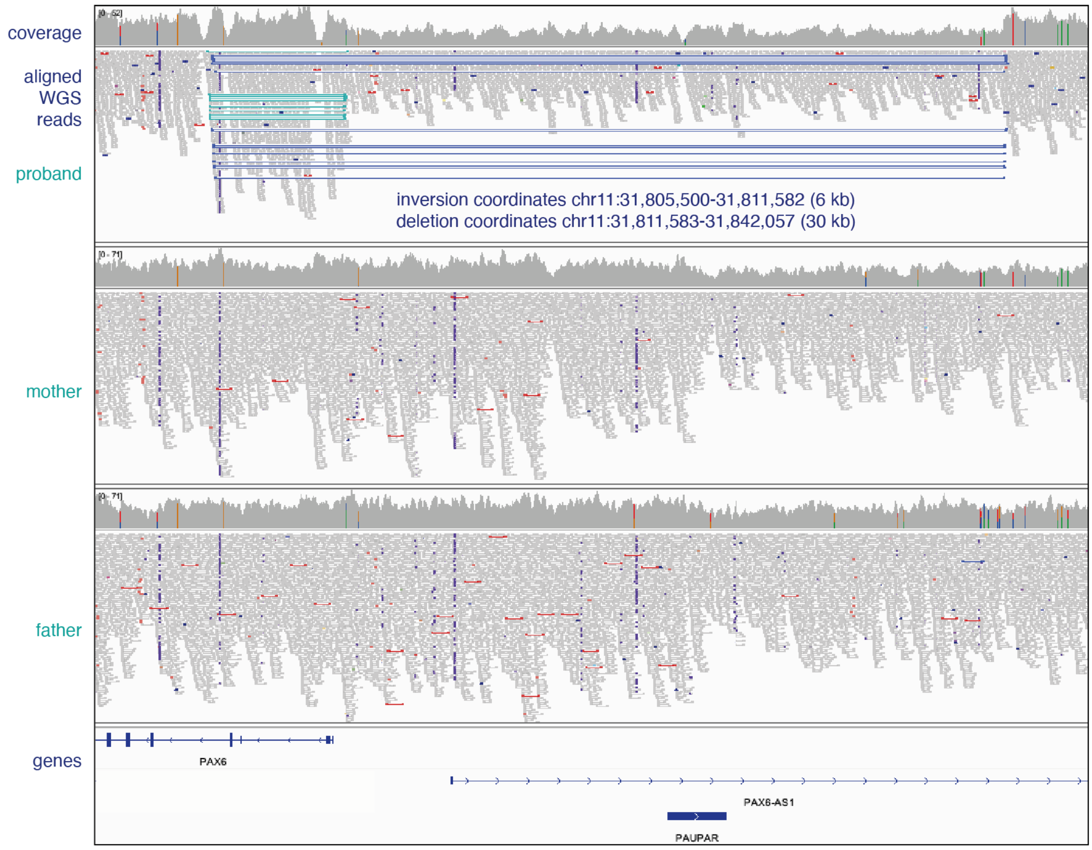

B Single proband RPID 1271: tandem duplication and deletion

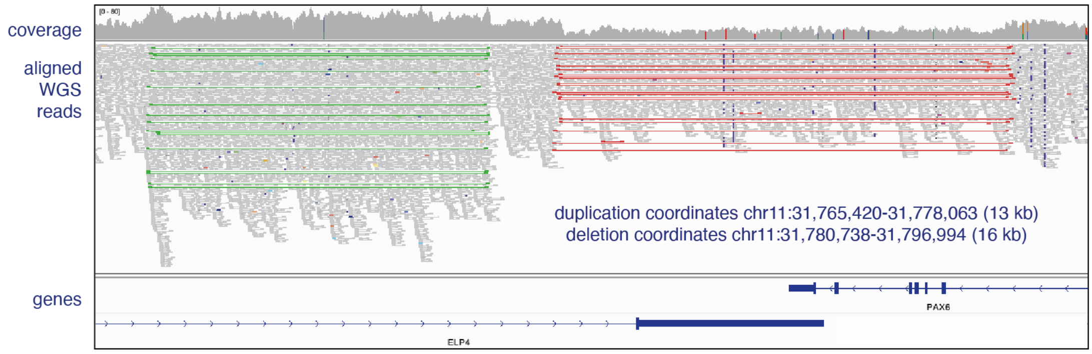

Supplementary Figure 12: Heterozygous deletions encompassing *FOXC1* detected on WGS. Aligned WGS reads shown with deletions in red; genomic coordinates are hg38. The coverage over the *FOXC1* gene itself was poor, as expected for an area of high GC content. (A) RPID 1142 (child with sporadic aniridia), 83 kb deletion, chr6:1,544,064-1,626,702. (B) RPID 1451 (child with aniridia presenting as congenital glaucoma, biscuspid aortic valve and mild aortic stenosis, 33 kb deletion, chr6:1,581,517-1,615,082. (C) RPID 1732 (a child with aniridia, glaucoma, and aortic stenosis), 82 kb deletion, chr6:1,604,452-1,686,063.

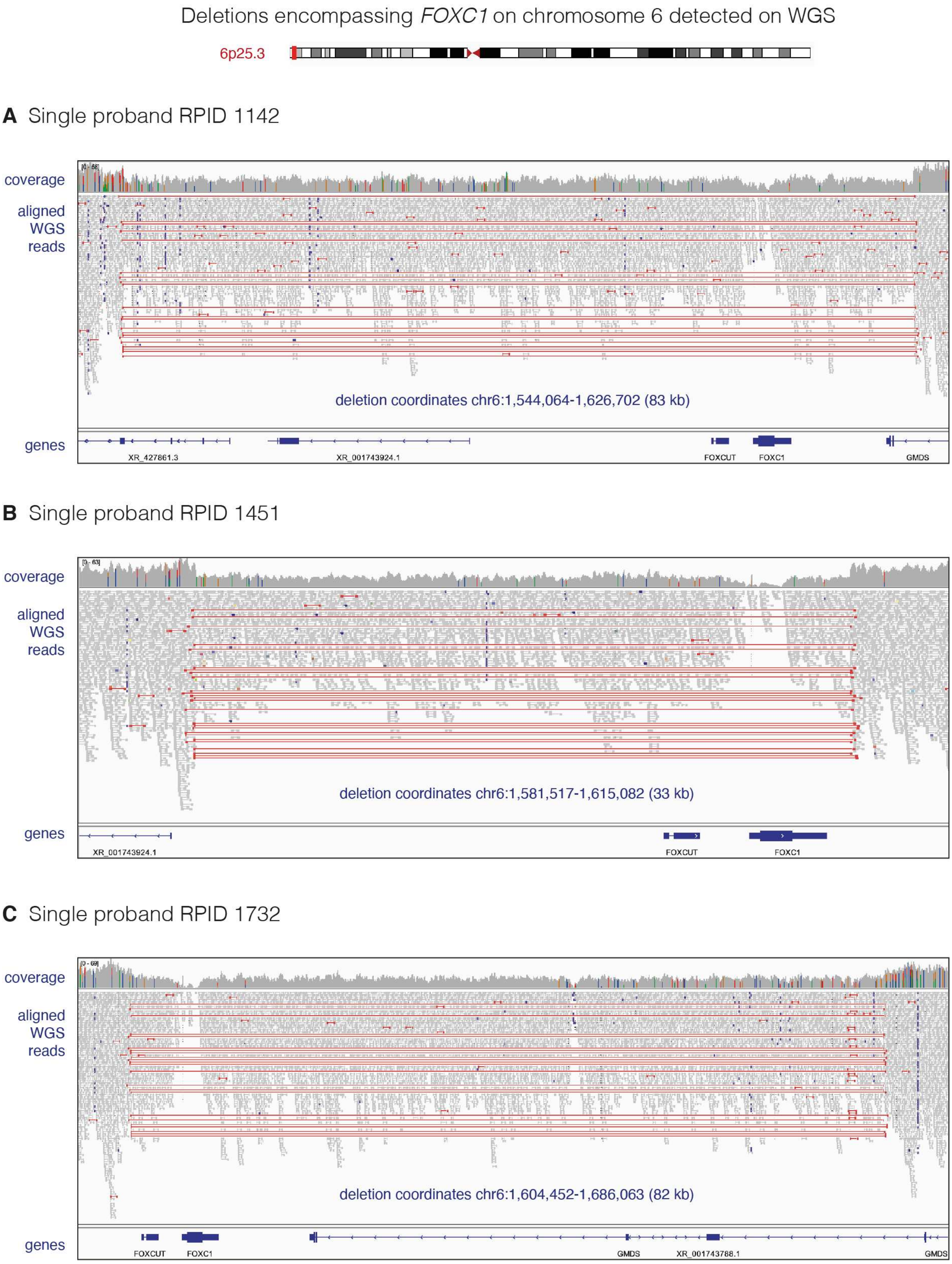
Deletions encompassing *FOXC1* on chromosome 6 detected on WGS
6p25.3
A Single proband RPID 1142
coverage
aligned WGS reads
deletion coordinates chr6:1,544,064-1,626,702 (83 kb)
genes
XR_427861.3
XR_001743924.1
FOXCUT
FOXC1
GMDS
B Single proband RPID 1451
coverage
aligned WGS reads
deletion coordinates chr6:1,581,517-1,615,082 (33 kb)
genes
XR_001743924.1
FOXCUT
FOXC1
C Single proband RPID 1732
coverage
aligned WGS reads
deletion coordinates chr6:1,604,452-1,686,063 (82 kb)
genes
FOXCUT
FOXC1
GMDS
XR_001743788.1
GMDS

Supplementary Figure 13: *De novo* reciprocal chromosomal translocation t(1,9) in RPID 356. (A) Ideogram before translocation showing intact chromosomes 1 and 9 with the areas of subsequent structural rearrangement marked out: 4.5 Mb paracentric inversion of chromosome 9 (orange) and t(1,9) translocation (blue). Each end of the rearranged segments are marked with a letter A-H for orientation purposes below. (B) Ideogram after structural rearrangement. (C) WGS data viewed in IGV (alignments coloured by insert size and pair orientation) for RPID 356 over each of the 3 breakpoints, the letters corresponding to those in the ideogram above. Data from the parents (not show) confirmed the translocation as *de novo*. The genomic sites of the breakpoint lie in the centre of the panel for each of the 3 breakpoints (blue arrow of top panel): as this is a heterozygous SV, approximately half the reads either side of the breakpoint are coloured to indicated that they have paired mate mapping to a different chromosomal location. Breakpoint B-C: chromosome 1 breakpoint, at chr1(hg38):21,560,758 (p36.12), in the *ALPL* gene, with the green-coloured reads either side of the breakpoint denoting paired mates mapping to two different areas of chromosome 9. The chromosome 9 breakpoints are labelled D-E, at chr9:89,372,728 (q22.1), and F-G, at chr9:93,847,511 (q22.31). The light purple reads on the left of each of these two panels indicates that the paired mate maps to chromosome 1; the blue reads on the right indication a difference in pair orientation consistent with the chromosome 9 inversion.

Reciprocal translocation t(1,9) in RPID 356 (likely benign)

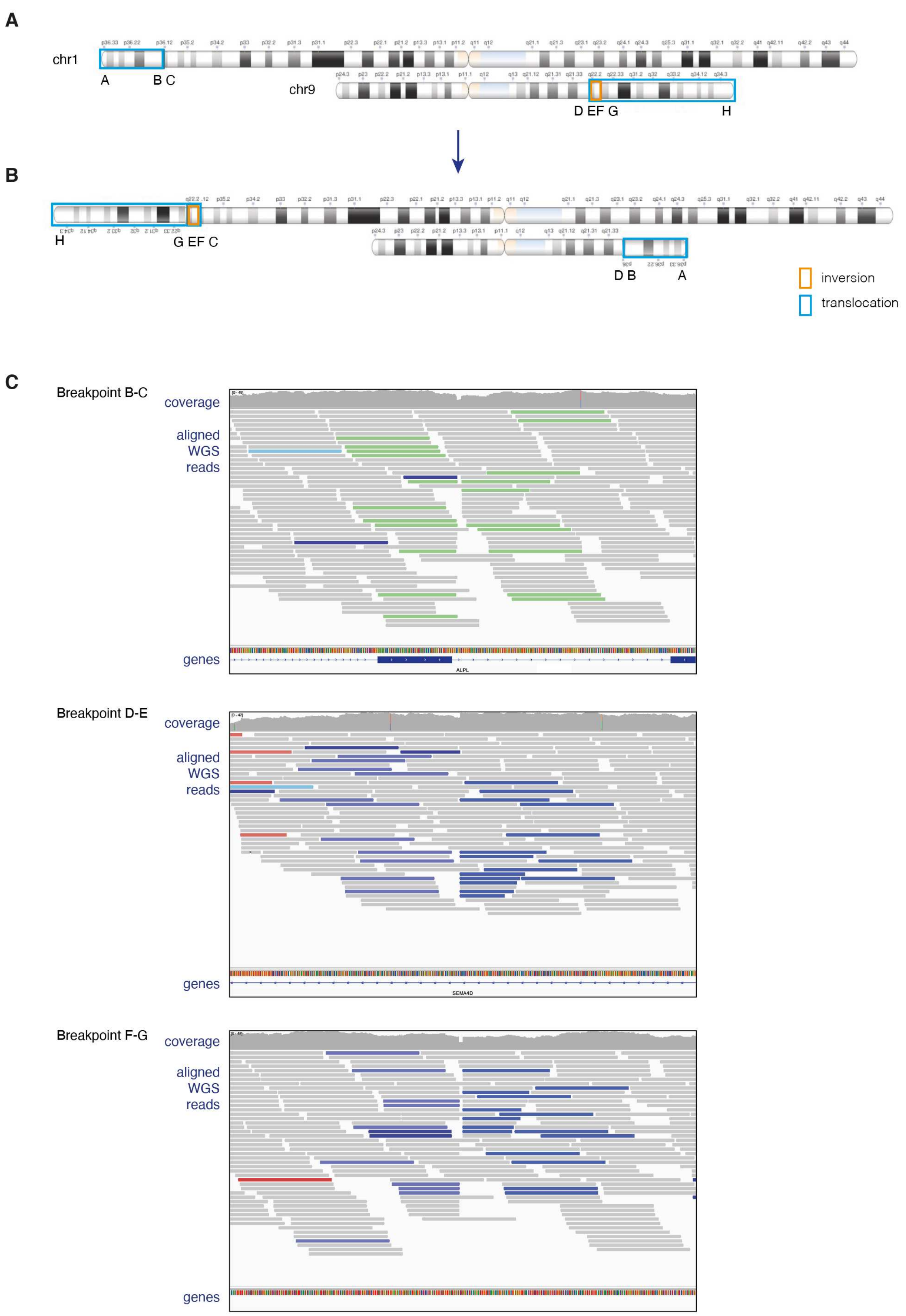

References

1. Bhatia S, Bengani H, Fish M, Brown A, Divizia MT, de Marco R, et al. Disruption of autoregulatory feedback by a mutation in a remote, ultraconserved PAX6 enhancer causes aniridia. Am J Hum Genet. 2013 Dec 5;93(6):1126–34.

2. Lappalainen I, Lopez J, Skipper L, Hefferon T, Spalding JD, Garner J, et al. dbVar and DGVa: public archives for genomic structural variation. Nucleic Acids Res. 2013 Jan 1;41(D1):D936–41.

3. Wang X, Wei X, Thijssen B, Das J, Lipkin SM, Yu H. Three-dimensional reconstruction of protein networks provides insight into human genetic disease. Nat Biotechnol. 2012 Jan 15;30(2):159–64.

4. Schmitt AD, Hu M, Jung I, Xu Z, Qiu Y, Tan CL, et al. A Compendium of Chromatin Contact Maps Reveals Spatially Active Regions in the Human Genome. Cell Rep. 2016 Nov;17(8):2042–59.

Supplementary Tables

for Hall *et al*, Short-read whole genome sequencing identifies causative variants in most individuals with previously unexplained aniridia

Supplementary Tables:

S1: Genomic regions examined on IGV for direct visualisation of SV breakpoints

S2: Nested primers used in RT-PCR splicing analysis

S3: STR profiling

S4: Splicing predictions

S5: CREs examined

S6: CREs examined with frequencies

S7: *De novo* variants

Table S1: Genomic regions examined on IGV for direct visualisation of SV breakpoints

Chromosome	Genomic coordinates (hg38)		Gene / Region	Notes
	Start	End		
**chr9**	18472082	18912949	ADAMTSL1	
**chr9**	93847400	93977000	BARX1 and regions translocation breakpoint/del	Not an aniridia gene. Included as was a breakpoint of the t(1,9) translocation in case RPID 356
**chr7**	129223015	129432211	AHCYL2	Not an aniridia gene. Included as *de novo* variant in this gene identified in RPID 2469.
**chr2**	38065603	38078181	CYP1B1	
**chr22**	45500839	45603134	FBLN1	
**chr6**	1395001	1615000	FOXC1 + 5' gene desert	
**chr1**	47414072	47420052	FOXE3	
**chr3**	4491348	4849840	ITPR1	
**chr3**	25426263	25599931	RARB	
**chr13**	35471789	35478695	MAB21L1	
**chr11**	31311354	32111353	PAX6 locus +5'/3' regulatory regions	
**chr11**	35660805	35811380	TRIM44	
**chr4**	110615801	111715800	PITX2 + 5' gene desert	

Table S2: Nested primers used in RT-PCR splicing analysis: oligonucleotide sequences

Primer name	Sequence 5' to 3'
Exon 1 to exon 5:	
**Green outer F (B1466)**	GCCAGTGAGGAGCGGTG
**Green outer R (B1468)**	CTGCAGAATTCGGGAAATG
**Green inner F (B1527)**	CACATTAACACACTTGAGCC
**Green inner R (B1463)**	TGTGAGCTAGCTCTACAATC
Exon 3 to exon 8:	
**Orange outer F (B1677)**	GCGCAGATGTGTGAGGCC
**Orange outer R (B1678)**	GTTGGAACTGATGGAGTTGG
**Orange inner F (B1675)**	GAGAGTGGACAGACATCCG
**Orange inner R (B1676)**	TCCTTCCTGTTGCTGGCAG
Exon 7 to exon 12:	
**Yellow outer F (B1523)**	AGCAACAGATGGGCGCAG
**Yellow outer R (B1524)**	ATTCACCGAAGGGCTGGTG
**Yellow inner F (B1525)**	GGCATGTATGATAAACTAAGG
**Yellow inner R (B1526)**	GCAGGAGTATGAGGAGGTC
Exon 9 to 3' UTR:	
**Pink outer F (B1681)**	GCCCGAGAAAGACTAGCAG
**Pink outer R (1682)**	CTGAAGCGGCTCTAACAGC
**Pink inner F (B1679)**	CAAAATAGATCTACCTGAAGC
**Pink inner R (B1680)**	TGTTGTGTCCCCATAGTCAC

Table S3: Short tandem repeat (STR) genotype profiling of family 2134 confirming that the two parental samples were the same at the 10 STR loci tested, and indicating (as suspected on WGS) that the "paternal" sample was in fact a duplicate of the maternal sample

Sample Name	Locus	Allele 1	Allele 2	Size 1	Size 2
**2134_2134_R45E2 Proband sample**	TH01	6	9	161.32	173.26
	D21S11	29	32	218.90	232.94
	D5S818	11	12	130.16	134.21
	D13S317	11		187.35	
	D7S820	11		232.03	
	D16S539	9		278.70	
	CSF1PO	11	12	337.24	341.33
	AMEL	X	Y	104.10	109.92
	vWA	14	18	138.56	154.36
	TPOX	8	11	268.76	280.75
**2134_2137_R2A10 Parental sample**	TH01	9	10	173.30	177.30
	D21S11	29	32	218.90	230.99
	D5S818	11	12	130.20	134.23
	D13S317	11		187.41	
	D7S820	10	11	228.09	232.08
	D16S539	9		278.61	
	CSF1PO	12		341.15	
	AMEL	X		104.20	
	vWA	15	18	142.39	154.36
	TPOX	11		280.75	
**2134_2138_R45F3 Parental sample**	TH01	9	10	154.43	173.27
	D21S11	29	32	218.95	231.03
	D5S818	11	12	130.16	134.21
	D13S317	11		187.34	
	D7S820	10	11	228.02	232.14
	D16S539	9		278.64	
	CSF1PO	12		341.18	
	AMEL	X		104.13	
	vWA	15	18	142.40	154.43
	TPOX	11		280.80	

Note: STR profile for 10 loci run using the GenePrint10 System (Promega). Allele binning based on size performed in GeneMapper 4.0. Where a single allele is listed, this is in the homozygous state. Each allele number is coloured for visualisation purposes

Table S4: Predicted effects on splicing of *PAX6* sequence variants identified in the WGS cohort

**CDS variant (PAX6 NM_000280.4)**	**RPID(s)**	**Region affected**	**Analysis range (Alamut)**	**Splice site**	Alamut **SSF [0-100]**	Alamut **MaxEnt [0-16]**	Alamut **NNSPLICE [0-1]**	Alamut **GeneSplicer [0-21]**	**Splicing predic-tion**	**SpliceAI delta score [0-1]**
c.-52+1G>C	877	essential splice site (IVS3+1)	c.-128-84 (intron 2) to c.-52+85 (intron 3)	c.-52 N	100.00 → -				donor loss	0.58
c.-52+1G>T	1019	essential splice site (IVS3+1)	c.-128-45 (intron 2) to c.-52+124 (intron 3)	c.-52 N	100.00 → -				donor loss	0.58
c.-128-2del	1500 + 5645	essential splice site (IVS2-2)	c.-128-124 (intron 2) to c.52+45 (intron 3)	c.-128 N	86.66 → -	10.74 → -	0.99 → -	11.42 → -	acceptor loss	0.65
c.682+68C>G	1635	deep intronic (IVS8+68)	c.644 (exon 8) to c.682+175 (intron 8)	c.682+67	- → 84.79	- → 8.39	- → 0.93	- → 2.09	donor gain	0.97
c.357+334G>A	3612	deep intronic (IVS6+334)	c.357+212 to c.358-248 (intron 6)	c.357+331	- → 71.80	1.32 → 7.33 (+456.7%)	- → 0.80		donor gain	0.46

CDS, coding sequence; RPID, research participant identifier; N, natural splice site; SSF, splice site finder
Thresholds: SSF ≥ 70, MaxEnt ≥ 0, NNSPLICE ≥ 0.4, GeneSplicer ≥ 0

Table S5: GRCh38 coordinates of *PAX6 cis*-regulatory elements

Chromosome	Start	Finish	CRE Name	No. Variants
**chr11**	31640399	31641876	E+180B	1
**chr11**	31640562	31641230	HS8B	1
**chr11**	31641320	31641927	HS8A	0
**chr11**	31649471	31650142	HS5	1
**chr11**	31655216	31655799	HS3	3
**chr11**	31655891	31656443	HS2	1
**chr11**	31664033	31664626	SIMO	1
**chr11**	31691131	31691766	E+120	1
**chr11**	31712697	31713381	E100	1
**chr11**	31763231	31763878	E60A	2
**chr11**	31763748	31765309	E60B	2
**chr11**	31803813	31804584	NRE	1
**chr11**	31804143	31804358	Ele4	0
**chr11**	31806399	31806399	PAX6_ATG	0
**chr11**	31810960	31811514	P1_promotor	0
**chr11**	31811160	31811217	E1E	0
**chr11**	31816261	31816745	0COE1	3
**chr11**	31817808	31817962	P0_promotor	0
**chr11**	31819763	31820175	P2_agCNE14	0
**chr11**	31821668	31821983	EE_agCNE13	0
**chr11**	31822082	31822520	P_agCNE12	0
**chr11**	31823784	31824857	Up-8_agCNE11	4
**chr11**	31825288	31825612	Up-9_agCNE10	1
**chr11**	31826303	31826903	Up-10_agCNE9	1
**chr11**	31870906	31871516	PE3_E-52	1
**chr11**	31874695	31875223	E-55C_agCNE8	1
**chr11**	31876017	31876485	E-55B_agCNE7	2
**chr11**	31877182	31877419	E-55A_agCNE6	2
**chr11**	31886918	31887337	E-72	2
**chr11**	31967909	31968253	Id855_agCNE5	0
**chr11**	31994909	31995566	agCNE4	3
**chr11**	32003264	32003431	agCNE3	0
**chr11**	32031078	32031697	E-200_agCNE2	1
**chr11**	32041336	32041684	agCNE1	3
**chr11**	32063979	32064758	E-250	1

Table S6: Variants called within *PAX6* CREs

Chromosome	Position	ID	Ref	Alt	AC	In gnomAD
**chr11**	31640570	rs116344813	C	T	1	Y
**chr11**	31641108	rs7480296	C	T	105	Y
**chr11**	31649995	rs75155674	A	G	3	Y
**chr11**	31655378	rs527750251	GAA	G,GA	3,55	Y
**chr11**	31655412	.	CT	C	1	Y
**chr11**	31655529	rs141010562	T	C	1	Y
**chr11**	31655957	rs546016853	T	C	1	Y
**chr11**	31664450	rs77294851	G	A	1	Y
**chr11**	31691356	rs2032490	C	T	72	Y
**chr11**	31713193	rs7926476	T	C	35	Y
**chr11**	31763753	.	C	CT	1	Y
**chr11**	31763865	rs113252042	G	A	1	Y
**chr11**	31765243	.	T	G	1	Y
**chr11**	31803970	rs694617	T	G	94	Y
**chr11**	31816401	rs1540318	C	T	13	Y
**chr11**	31816580	rs4440995	G	A	13	Y
**chr11**	31816704	rs5790869	C	CG	106	Y
**chr11**	31823785	rs1805270	G	T	13	Y
**chr11**	31823821	.	C	G	1	Y
**chr11**	31824099	rs1806172	A	G	3	Y
**chr11**	31824409	rs35186763	CT	C,CTT	16,4	Y
**chr11**	31825436	.	C	CT	2	Y
**chr11**	31826416	rs370398382	TA	T	1	Y
**chr11**	31870966	rs662155	C	T	86	Y
**chr11**	31875104	rs564983219	C	A	1	Y
**chr11**	31876300	rs72896305	G	A	20	Y
**chr11**	31876337	rs140066452	C	T	1	Y
**chr11**	31877325	rs746615173	C	T	1	Y
**chr11**	31877339	rs11031506	T	C	40	Y
**chr11**	31887106	.	C	G	1	N
**chr11**	31887179	rs11031520	G	T	18	Y
**chr11**	31995087	rs224674	T	G	12	Y
**chr11**	31995236	rs11031547	T	C	11	Y
**chr11**	31995530	rs224673	T	C	12	Y
**chr11**	32031461	rs1002229	A	T	9	Y
**chr11**	32041474	rs193052496	A	G	1	Y
**chr11**	32041530	rs224611	T	C	50	Y
**chr11**	32041683	rs224612	T	C	75	Y
**chr11**	32064251	rs7128319	G	A	3	Y

BMJ Publishing Group Limited (BMJ) disclaims all liability and responsibility arising from any reliance placed on this supplemental material which has been supplied by the author(s)

Table S7: *De novo* analysis results of trio whole genomes in the aniridia cohort

**Individual (RPID)**	**Gene**	**Region**	**Chr**	**Genomic coordinates (hg38)**	**CDS variant**	**Protein / splicing consequence**	**CADD/ REVEL score**	**ACMG/ACGS**	**Novel**	**Comment**
75	*PRDM5*	Exon 16/16	4	g.120695125C>T	NM_018699.4: c.1879G>A	p.(Val627Ile)	22.6		yes	***De novoPAX6*** **SV identified**
356	*CRYBG2*	Exon 3/22	1	g.26354223_26354233del	ENST00000475866.3:c.729_739del	p.(Ala244Leufs*4)	n/a		yes	***De novoPAX6*** **SV identified**
1635	***PAX6***	**IVS8+68**	**11**	**g.31794562G>C**	**NM_000280.4: c.682+68C>G**	**p.(?) / donor gain**	**19.83**	**P (0.994)**	**yes**	**disrupts splicing *in vitro(2)***
	NYNRIN	Exon 9/9	14	g.24416718_24416719dup	NM_025081.3: c.4969_4970dup	p.(Ala1658Argfs*19)	34		no	trumped by *PAX6* variant above
2469	*AHCYL2*	Exon 8/17	7	g.129405125 C>A	NM_015328.4: c.1054C>A	p.(Leu352Met)	25 / 0.73	U (0.5)	yes	interacts with ITPR1
	IGLV3-1	Exon 2/2	22	g.22881377 G>A	ENST00000390319.2:c.327G>A	p.(Trp109*)	35		no	encodes a Bence-Jones protein
	ZBTB11	Exon 2/11	3	g.101671997 G>T	NM_014415.4: c.527C>A	p.(Ser176 Tyr)	25.8		yes	AR intellectual disability gene
	H3C4	Exon 1/1	6	g.26197043 G>T	NM_001376937.1:c.208C>A	p.(Arg70Ser)	23		yes	
	ZNF74	Exon 5/5	22	g.20406879_ 20406880ins GGGATCCCA	NM_003426.4: c.1846_1847ins GGGATCCCA	p.(Pro615_Ile616ins ArgAspPro)	n/a		yes	
**5645**	***PAX6***	**IVS2-2**	**11**	**g.31,806,927del**	**NM_015328.4: c.-128-2del**	**p.(?) / acceptor loss**	**24.4**	**LP (0.9)**	**no (3)**	**canonical splice variant**

Abbreviations: subs, substitution; del, deletion; dup, duplication; ins, insertion; IVS, intervening sequence/intron; CDS, coding sequence; P, pathogenic; U, uncertain; LP, likely pathogenic (all ACMG/ACGS criteria as previously)
